# Systematic functional evaluation of *CNGA1* missense variants associated with retinitis pigmentosa

**DOI:** 10.1186/s10020-026-01518-0

**Published:** 2026-05-27

**Authors:** Peggy Reuter, Jennifer Schroeder, Marc Sturm, Mathieu Quinodoz, Veronika Vaclavik, Miriam Bauwens, Marieke De Bruyne, Bart Leroy, Joseph van Aerschot, Katarina Stingl, Susanne Kohl

**Affiliations:** 1 Institute for Ophthalmic Research, Centre for Ophthalmology, University Hospital Tübingen, Elfriede-Aulhorn-Str. 7, Tübingen, D-72076 Germany; 2 Institute of Medical Genetics and Applied Genomics, University of Tübingen, Tübingen, 72076 Germany; 3 Institute of Molecular and Clinical Ophthalmology Basel (IOB), Basel, 4031 Switzerland; 4 Department of Ophthalmology, University of Basel, Basel, 4031 Switzerland; 5 Jules-Gonin Eye Hospital, Fondation Asile des Aveugles, University of Lausanne, Lausanne, 1004 Switzerland; 6 Department of Biomolecular Medicine, Ghent University, Ghent, Belgium; 7 Center for Medical Genetics, Ghent University Hospital, Ghent, Belgium; 8 Department of Head and Skin, Ghent University, Ghent, Belgium; 9 Department of Ophthalmology, University Hospital Leuven, Louvain, Belgium; 10 University Eye Hospital, Center for Ophthalmology, University of Tübingen, Tübingen, 72076 Germany

**Keywords:** *CNGA1*, Variants of uncertain significance, Functional evaluation, Retinitis pigmentosa, Inherited retinal disease

## Abstract

**Background:**

Missense variants are frequently classified as variants of uncertain significance (VUS) according to the guidelines of the American College of Medical Genetics and Genomics and the Association of Molecular Pathology (ACMG/AMP). Consequently, disease relevance remains elusive, impeding molecular genetic diagnostics, patients` and family genetic counseling, and identification of patients eligible for clinical trials. Functional studies are critical for resolving the clinical significance of VUS. *CNGA1* encodes the main subunit of the rod cyclic nucleotide-gated (CNG) channel, a vital component of the phototransduction cascade. Variants in *CNGA1* are a rare cause of autosomal recessive retinitis pigmentosa and a phase I/II gene augmentation trial (NCT06291935) is currently ongoing highlighting the necessity to differentiate benign from pathogenic variants.

**Methods:**

*CNGA1* missense variants compiled from retinal disease patient cohorts, public databases and literature were functionally investigated using a medium-throughput aequorin-based assay and in vitro minigene splice assays for predicted exonic spliceogenic variants. Functional data were correlated with the in silico prediction of five variant effect predictors (VEPs) and applied to support or revise variants’ ACMG/AMP classification.

**Results:**

Data mining revealed 86 missense *CNGA1* variants – including three novel – most of them lacking functional data; 65.1% of the variants were initially classified as VUS. The aequorin-based assay showed that 72.1% of tested variants significantly impaired CNG channel function and were classified as functionally abnormal, while 23.3% were functionally normal and 5% remained functionally uncertain. Correlation of the functional data with in silico predictions identified AlphaMissense and CPT-1 to be the most suitable tools for assessing *CNGA1* missense variants. Using in vitro minigene splice assays, two putative missense variants were shown to induce missplicing. Based on the functional findings, 62.1% of the variants initially classified as VUS were re-categorized as likely pathogenic or likely benign. Furthermore, 93.3% of the variants initially classified as likely pathogenic showed an effect on CNGA1 channel function, confirming their disease relevance and supporting their reclassification as pathogenic.

**Conclusion:**

This study represents the first comprehensive functional assessment of disease-associated *CNGA1* missense variants, thus significantly advancing the understanding of their disease relevance and improving molecular genetic diagnostics in patients.

**Supplementary Information:**

The online version contains supplementary material available at 10.1186/s10020-026-01518-0.

## Background

Retinitis pigmentosa (RP; OMIM #268,000) is the most common inherited retinal dystrophy (IRD) with a prevalence of 1:4,000 (Hartong et al. 2006). RP is genetically highly heterogeneous, and to date, more than 100 RP-associated genes have been identified, most of which (80%) are linked to autosomal recessive inheritance (Rivolta et al. 2025); https://retigene.erdc.info/; accessed November 2025). Clinically, RP is characterized by progressive retinal degeneration that initially affects rod photoreceptor function and later also impairs cone function. Patients develop night blindness, followed by progressive visual field constriction and eventual blindness in advanced stages (Fahim et al. 1993). Variants in *CNGA1* have been identified as a rare cause of autosomal recessive RP (Dryja et al. 1995).

*CNGA1* encodes for the main subunit of the rod cyclic-nucleotide gated (CNG) channel – a ligand-gated non-selective cation channel which is an essential component of the phototransduction cascade in rod outer segments (Kaupp et al. 1989). Rod CNG channels are heterotetrameric channels composed of CNGA1 and CNGB1 subunits with a stoichiometry of 3A:1B (Shuart et al. 2011; Xue et al. 2022). The CNGA1 subunit is composed of six transmembrane domains (TD), a pore forming region between TD5 and TD6 and a C-linker that connects the cyclic nucleotide-binding domain (CNBD) with TD6. Downstream of the CNBD, CNGA1 also possesses a C-terminal leucine zipper (CLZ) domain essential for subunit assembly (Shuart et al. 2011). Early studies on bovine rod outer segments indicated that in the retina, the N-terminal region of CNGA1 is cleaved (Molday et al. 1991). CNGA1 represents the main subunit of the rod CNG channel whereas CNGB1 represents the modulatory subunit influencing e.g. the apparent ligand sensitivity and modulation capability by calmodulin (Chen et al. 1993; Korschen et al. 1995; Weitz et al. 1998). The CNGB1 subunit is also known to be essential for proper rod outer segment trafficking of the native rod CNG channel (Kizhatil et al. 2009; Pearring et al. 2021).

To date, 81 disease-associated *CNGA1* variants are listed in the Human Gene Mutation Database (HGMD®pro; accessed June 2025) – almost half (46%) being missense variants. To evaluate the disease relevance of variants, the American College of Medical Genetics and Genomics (ACMG) and the Association of Molecular Pathology (AMP) established an unbiased classification framework based on five categories ranging from benign (B), likely benign (LB), variant of uncertain significance (VUS), likely pathogenic (LP) to pathogenic (P) (Richards et al. 2015). Criteria for variant evaluation include e.g. variant frequency as well as presence/absence of homozygous variant carriers in the general population, presence of a second (L)P variant for an autosomal recessive disease, allelic data or segregation information, and in silico prediction of variant effects on protein or transcript level. Evaluation of nonsense and frameshift variants or variants affecting canonical splice sites is often straightforward using the ACMG/AMP classification system, as many of these variants are classified as (L)P (e.g., if loss-of-function is a known disease mechanism). Missense variants and candidate spliceogenic variants outside the canonical dinucleotides are more challenging to assess, and are often categorized as VUS indicating that their disease relevance remains elusive. In patients carrying one or two VUS, the cause of an autosomal recessive disease can therefore not be confirmed at the molecular genetic level. This hampers first of all genetic counseling of such patients and their families, but also impacts the development of therapeutic concepts.

The urge to provide reliable classification of *CNGA1* variants was increased, when the first-in man clinical trial was started at our center to assess the safety and efficacy of a gene supplementation approach for patients with *CNGA1*-associated RP (NCT06291935). Patients carrying *CNGA1* VUS are typically not eligible for clinical trials, as these studies rely on small patient cohorts and inclusion of individuals with an uncertain or incorrect genetic diagnosis may compromise study outcomes, since they are unlikely to benefit from gene-specific therapies. In addition, *CNGA1*-associated RP is rare, and the pool of eligible patients is further reduced by the presence of VUS.

Disease association of VUS can be clarified by functional data enabling ACMG/AMP-based classification of variants into categories (L)B or (L)P (Brnich et al. 2019). Candidate spliceogenic variants in IRD genes – which are mostly not expressed in accessible patient tissue such as blood – are commonly studied using in vitro minigene splice assays (Sangermano et al. 2018; Westin et al. 2021; Reuter et al. 2023; Rawnsley et al. 2025). The evaluation of missense variants is usually more challenging: functional approaches have e.g. extensively been applied for the evaluation of variants in *BRCA2* associated with an increased risk of early-onset breast and ovarian cancer (Guidugli et al. 2013; Ikegami et al. 2020; Huang et al. 2025), but only a limited number of medium- or high-throughput approaches have been performed to assess missense variant effects in IRD genes e.g. in *RHO*, *CNGA3* or *CRX* (Wan et al. 2019; Solaki et al. 2023; Shepherdson et al. 2024). Typically, only single or few missense variants are functionally characterized as exemplified by studies on *RPE65* or *ABCA4* (Lorenz et al. 2008; Philp et al. 2009; Soens et al. 2017; Garces et al. 2018; Azizzadeh Pormehr et al. 2025).

Over the last decades, CNGA1 has been used extensively to study different aspects of CNG channel structure and function, e.g. ligand binding, ion conductivity/selectivity, gating and subunit assembly (Becchetti & Gamel 1999; Mazzolini et al. 2002, 2018; Zhong et al. 2003; Kusch et al. 2004). Preferentially, bovine CNGA1 was heterologously expressed in *Xenopus laevis* oocytes or standard cell lines and often combined with scanning mutagenesis approaches to elucidate the importance of individual amino acid residues for CNGA1 function. Unfortunately, functional and/or immunocytochemical data for human disease-associated *CNGA1* variants are only available for a few variants (Dryja et al. 1995; Gao et al. 2019). Therefore, we used our well-established medium-throughput aequorin-based luminescence assay (Tager et al. 2018, 2021; Kohl et al. 2021; Solaki et al. 2023) to functionally evaluate all currently documented RP- or IRD-associated *CNGA1* missense variants within functionally and structurally important channel domains. In addition, selected candidate spliceogenic exonic *CNGA1* variants were investigated using in vitro minigene splice assays. The obtained functional readout was applied to the ACMG/AMP classification of these variants to confirm or dismiss their disease relevance.

## Methods

### *CNGA1* transcript analysis

To identify and confirm the *CNGA1* transcript predominantly expressed in human retina, RNA-seq data of four post-mortem human retina samples from the ArrayExpress dataset E-MTAB-4377 were studied (Pinelli et al. 2016). The number of spliced reads per exon-exon junction was determined and set in relation to the three *CNGA1* transcripts NM_001379270.1, NM_000087.5 and NM_001142564.1. *CNGA1* transcription start sites (TSS) were assessed using cap analysis of gene expression sequencing (CAGE-seq) data from the FANTOM5 consortium (Forrest et al. 2014; Lizio et al. 2019). To assess *CNGA1* transcript isoforms, the alternative splicing catalog of the transcriptome (ASCOT) data set (http://ascot.cs.jhu.edu/ucsctracks.html) which comprises human RNA-seq data from 53 tissues (originating from the Genotype-Tissue Expression (GTEx) project) supplemented with RNA-seq data from peripheral retina (Ling et al. 2020) was accessed via the UCSC (University of California Santa Cruz) genome browser (http://genome.ucsc.edu/). As *CNGA1* showed strong expression in the retina and only low expression within the 53 tissues of the GTEx set, tracks of the liver, nerve and adipose-subcutaneous tissue were selected as representative examples for *CNGA1* expression in non-retinal tissue.

### *CNGA1* variant selection and ACMG/AMP classification

All *CNGA1* missense variants located in functionally/structurally important protein domains and being associated with RP or IRD were retrieved from the *CNGA1*-RP study group which comprises the local Tübingen RetDis database, the genetic databases of the Center for Medical Genetics of the Ghent University, Belgium, and the Institute of Molecular and Clinical Ophthalmology of the University of Basel, Switzerland, and from public databases, i.e. the Leiden Open Variation Database (LOVD; https://www.lovd.nl/), ClinVar (https://www.ncbi.nlm.nih.gov/clinvar/), the Human Gene Mutation Database (HGMDpro) queried in May 2024 or from literature. All *CNGA1* variants were carefully (re-)annotated to the MANEselect transcript NM_001142564.1.

*CNGA1* missense variants were semi-automatically classified following ACMG/AMP guidelines (Richards et al. 2015) applying the web-based variant interpretation tool Franklin (Genoox Ltd, https://franklin.genoox.com/) and manual adaptation of the applied criteria. The criterion BA1 was applied for variants with a grpmax filtering allele frequency (AF) > 5% in the gnomAD v4.1.0 (non-UKB) dataset (https://gnomad.broadinstitute.org/). The threshold for application of the criterion BS1 (grpmax filtering AF > 0.00297) was calculated using the online tool https://cardiodb.org/allelefrequencyapp/ (Whiffin et al. 2017) under consideration of a RP prevalence of 1:4,000, a genetic heterogeneity of 0.08, an allelic heterogeneity of 0.63 and a penetrance of 0.9 (Katagiri et al. 2014; Suga et al. 2022). PM3 was applied for variants with PopMax AF < 0.0022%. BS2 was employed for variants being detected in > 2 homozygotes in the general population in the gnomAD v4.1.0 (non-UKB) dataset. For application of the criterion PP3/BP4, the (1) REVEL scores (Ioannidis et al. 2016) retrieved using dbNSFP v5.1a (https://www.dbnsfp.org/) (Liu et al. 2011, 2020) were considered and criterion strength was manually adapted following the suggestions from Pejaver and colleagues (Pejaver et al. 2022) and (2) SpliceAI scores (https://spliceailookup.broadinstitute.org/) were evaluated and PP3_supporting was implemented for a SpliceAI score ≥ 0.2 (Abou Tayoun et al. 2018; Walker et al. 2023). BP4 was applied only if SpliceAI score ≤ 0.1 and REVEL score ≤ 0.29. The criteria BP6/PP5 were not considered for variant classification (Biesecker et al. 2018). All *CNGA1* variants were initially classified without any functional data and subsequently re-classified applying the data obtained from the aequorin-based bioassay. Following the recommendation of Brnich et al., the likelihood ratio of pathogenicity (OddsPath score) for benign and pathogenic variants was calculated based on control variants included in the aequorin-based bioassay, enabling application of the criterion PS3_strong for “functionally abnormal” variants and the criterion BS3_supporting for “functionally normal” variants (Brnich et al. 2019; Muhammad et al. 2024). For variants with uncertain functional evaluation or variants locating within the CLZ domain neither criterion was applied. Application of the in vitro minigene splice data followed the recommendations of Abou Tayoun and colleagues (Abou Tayoun et al. 2018).

Three variants with a clinical significance of “benign” were retrieved from the gnomADv4.1.0 (non-UKB) dataset based on a grpmax filtering AF ≥ 0.01 and ≥ 15 homozygotes in the general population and used for assay validation.

### Evolutionary conservation of the human CNGA1 subunit

The protein sequence alignment of CNGA1 and CNGA3 subunits from rod and cone CNG channels from six different species (Supplementary Table 1) was generated using ClustalOMEGA (https://www.ebi.ac.uk/jdispatcher/msa/clustalo) with default settings also enabling calculation of the protein sequence identity. Visualization of the alignment and color-coding based on sequence identity was done with JalView (Waterhouse et al. 2009) (https://www.jalview.org/) using 50% identity as threshold.

### Generation of CNGA1/3 variant expression constructs

To test *CNGA1* missense variants in the CNGA3-based assay, the homologous CNGA1/CNGA3 residues were determined by ClustalOMEGA alignment (Supplementary Fig. 1) and mutated to match the CNGA1 variants. When the native CNGA1 and CNGA3 residues differed, the CNGA3 residue was additionally changed to the CNGA1 wild-type residue. These are referred to as CNGA1/A3 homology control variants. The wild-type *CNGA3* expression construct was generated as described previously (Reuter et al. 2008; Koeppen et al. 2008) and served as template for generation of mutant expression plasmids via in vitro mutagenesis (IVM) PCR using the PfuUltra High-Fidelity DNA Polymerase (Agilent; Santa Clara, CA, USA) and following the Quick-change site directed mutagenesis protocol from Agilent (Santa Clara, CA, USA). For IVM, PCR primers were codon-optimized when the targeted homologous *CNGA3* variant could not be generated by a single nucleotide substitution, and are provided in Supplementary Table 2. Mutant plasmids were verified by Sanger sequencing applying the Supre dye v3.1 cycle sequencing kit (AdvancedSeq LLC, Livermore, CA, USA) and covering the promoter, the *CNGA3* insert and the BGH poly terminator site.

### HEK293 cell cultivation and expression of modeled CNGA1 variants

Human embryonic kidney cells stably expressing the calcium-sensitive photoprotein apo-aequorin (referred to as HEK293^aequo^ cells) were used as heterologous expression system to study the CNGA1 variants. Cultivation and transfection of the HEK293^aequo^ cells for the aequorin-bioassay and for isolation of protein lysates for dot blot analysis was performed as previously described (Solaki et al. 2023).

### Aequorin-based bioassay

The aequorin-based bioassay was performed as previously described (Solaki et al. 2023). Untransfected HEK293^aequo^ cells and cells expressing the wild-type CNGA3 channel served as controls. Per variant at least three independent biological replicates (transfections) with three technical replicates each were studied.

### Data analysis

Luminescence data were analyzed using the MARS Data Analysis Software 3.42R5 (BMG LABTECH GmbH, Ortenberg, Germany). For each biological replicate per variant or control, the average of the technical replicates was calculated and the area under the curve (AUC, overall luminescence intensity) was computed for the luminescence signal for a time window of 279 s starting 14 s after the application of 8-bromoguanosine 3’,5’-cyclic monophosphate (8-Br-cGMP). In addition, the signal latency was evaluated, representing the time after measurement onset until the maximal luminescence response was reached. The AUC and the latency of the untransfected control and the CNGA1 variants were normalized to the wild-type CNGA3 response present on the same plate. Data of the biological replicates were averaged. Z-score based data analysis was performed to differentiate functionally normal and abnormal responses based on the control group comprising missense variants classified with a clinical significance of benign on gnomAD and the CNGA1/A3 homology controls. Based on this control group, the mean (mean_control_) and standard deviation (SD_control_) was calculated for the AUC and latency. Z-score based thresholds were defined as mean_control_ ± 2.56xSD_control_ for AUC and latency and corresponded to a statistical significance of p ≥ 0.01. Combining the thresholds for AUC and latency, variants were categorized as functionally normal, abnormal or uncertain. For functionally normal variants the mean ± SD located within the thresholds for latency and AUC. Variants were classified as “functionally abnormal” if their mean ± SD for AUC or latency were below or above the thresholds. Variants for which the mean ± SD of both parameters overlapped with the thresholds, or one parameter overlapped and the second parameter was within the thresholds, were categorized as “functionally uncertain”.

Identification of CNGA1 variants showing no response (from now on referred to as non-functional) was based on the average luminescence intensity prior to and after the addition of 8-Br-cGMP and manual revision. For variants that did not show a luminescence response, as well as variants with a normalized AUC < 0.02, the latency was set to zero.

### Dot blot analysis to confirm expression of CNGA1 variant channels

Preparation of whole-cell lysates, total protein extraction and dot blots analysis were performed as previously described (Solaki et al. 2023). Whole cell lysates from the biological replicates were pooled prior to total protein extraction. Untransfected HEK293^aequo^ cells and cells expressing the CNGA3_wt_ served as control.

### Generation of wild-type and mutant *CNGA1* minigene constructs

Wild-type and mutant minigene vectors comprising *CNGA1* exons 7 to 10 were generated as previously described (Weisschuh et al. 2012; Rawnsley et al. 2025). To evaluate a *CNGA1* variant locating in the last exon (exon 11), a modified pSPL3 vector was applied. Using inverse PCR and PfuUltra High-Fidelity DNA Polymerase (Agilent, Santa Clara, CA, USA), the splice acceptor exon was deleted from the pSPL3 vector; the new vector is designated as pSPL3_acc-del (see Supplementary Table 3). *CNGA1* fragments spanning exons 7 to 10 or exons 10 to 11, including up- and downstream flanking intronic sequence (250–300 bp), were PCR amplified from genomic DNA of a healthy donor using the ALLin™ HiFi DNA polymerase. Primers used for *CNGA1* fragment amplification contained restriction enzyme sites for subsequent cloning of the PCR products into the pSPL3 or pSPL3_acc-del vector and are listed in Supplementary Table 3. Based on these wild-type constructs, mutant minigenes were established via IVM PCR as described above using primers summarized in Supplementary Table 3. Exon–intron junctions of the wild-type and mutant minigenes were verified by Sanger sequencing.

### In vitro minigene splice assay

To perform in vitro minigene splice assays, human embryonic kidney cells (HEK293T/17; ATCC® CRL-11268™) (https://www.atcc.org, Manassas, VA, USA) were cultured and transfected as described previously (Rawnsley et al. 2025). Cells transfected with the empty pSPL3 or pSPL3_acc-del vectors and untransfected cells served as controls. Twenty-four hours post transfection, total RNA was isolated using the GenUP™ Total RNA Kit (biotechrabbit GmbH, Berlin, Germany). To evaluate, wild-type and mutant minigene splice products, 1 µg of DNase-treated total RNA was reverse transcribed into complementary DNA (cDNA) using either the pSPL3 transcript-specific primer 5’-ATCTCAGTGGTATTTGTGAGC-3’ or Oligo d(T) primer as described previously using the OneScript®Plus cDNA Synthesis Kit Applied Biological Materials, Vancouver, Canada) (Rawnsley et al. 2025). Primers used for reverse transcription (RT-)PCRs are listed in Supplementary Table 3. Two independent transfection and cDNA synthesis were performed. Quantification of splice products from mutant and wild-type CNGA1_ex7-10 mg was performed on agarose gel images using ImageJ (Schneider et al. 2012).

### In silico missense variant effect prediction and correlation with the functional bioassay data

For CNGA1 missense variants outside the CLZ domain, the functional data from the aequorin-based bioassay were correlated with the in silico prediction of five different variant effect predictors (VEP). Scores for REVEL, PrimateAI and CADD_Phred (CADDv1.7) were retrieved using dbNSFP v5.1a (https://www.dbnsfp.org/) (Liu et al. 2011, 2020). For these three VEPs, the threshold scores for categorizing a variant as P or B were determined according to the suggestions of Pejaver and colleagues (Pejaver et al. 2022). AlphaMissense scores were obtained from https://alphamissense.hegelab.org/ and variants with scores < 0.34 were considered LB and variants with scores > 0.564 as LP as suggested by AlphaMissense (https://alphamissense.hegelab.org/). Scores for CPT-1 were retrieved from https://zenodo.org/records/8140323 (Jagota et al. 2023). As threshold to differentiate P and B variants were not available, a receiver operating characteristic (ROC) analysis was performed based on the CPT-1 scores for functionally normal and abnormal CNGA1 variants using GraphPad Prims 10.1.1 (GraphPad Software, Boston, MA, USA). For all VEPs, the higher the score, the higher the probability that a variant is pathogenic – for REVEL, AlphaMissense, PrimateAI and CPT-1 the scores range from 0 to 1 and for CADD_Phred from 0 to 99. To assess the overall performance of the VEPs, the fraction of functionally normal variants correctly classified as LB or erroneously classified as LP as well as the fraction of functionally abnormal variants correctly classified as LP or erroneously categorized as LB was determined. To correlate the functional data and the VEP scores, the criteria AUC and latency (L) studied with the aequorin-based bioassay were combined into one functional score for each CNGA1 variant studied. The AUC was min–max normalized using the equation

$${AUC}_{n}=(AUC-{AUC}_{min})/({AUC}_{max}-{AUC}_{min})$$

where ${AUC}_{min}$ is the smallest AUC and ${AUC}_{max}$ the highest AUC value in the dataset. The latency was also min–max normalized applying the following two equations:

$$\mathrm{F}\mathrm{o}\mathrm{r} \mathrm{L}_{\mathrm{m}\mathrm{i}\mathrm{n}}\le \mathrm{L}\le 1: {L}_{n}=(L-{L}_{min})/(1-{L}_{min})$$

$$\mathrm{F}\mathrm{o}\mathrm{r} 1<\mathrm{L}\le \mathrm{L}_{\mathrm{m}\mathrm{a}\mathrm{x}}:{L}_{n}=(L-1)/({L}_{max}-1)$$

where ${L}_{min}$ is the smallest latency value and ${L}_{max}$ the highest latency value in the dataset. To calculate the functional score (FC), the normalized parameters AUC_n_ and L_n_ were linearly combined using the subsequent equation:

$$FC={AUC}_{n}\times 0.5+{L}_{n}\times 0.5$$

Equal weighting of both parameters was chosen since the luminescence reaction reflects a combination of the CNG channel's biophysical and biological properties (e.g. apparent ligand sensitivity, surface expression, ion selectivity and calcium conductance), and variants often impair different channel characteristics, affecting the AUC and/or latency (Solaki et al. 2023). The Spearman correlation coefficients of the functional scores and the VEP scores were calculated using GraphPad Prims 10.1.1.

## Results

### Identification of the main *CNGA1* transcript in the human retina

For human *CNGA1*, three different full-length transcripts are provided on public databases, e.g. NCBI (National Center for Biotechnology Information) and UCSC genome browser. Currently, 13 exons have been annotated for *CNGA1*, of which the first five are non-coding (exons 1, 1b, 2, 3 and 3b; Supplementary Fig. 2). To identify the *CNGA1* transcript predominantly expressed in the human retina, RNA-seq data of post-mortem human retina samples available from public databases were analyzed. A key observation was that the spliced read counts spanning exon 1/1b to exon 2 and exon 2 to exon 3 were 13- to 210-fold lower compared to counts spanning the subsequent canonically spliced exons (nomenclature refers to the MANEselect transcript NM_001379270.1; Supplementary Fig. 2). Alternative splicing was observed for the coding exons 7, 9 and 10, although the biological relevance is unclear (Supplementary Fig. 2), and for the non-coding exons 3/3b within the 5′ untranslated region (UTR) of *CNGA1*: (1) exon 3b was included in 2.9% of the spliced reads starting in exon 3 and (2) the majority of spliced reads (86.2%) starting in exon 2 showed skipping of exon 3 (and 3b) (Supplementary Fig. 2). In line with this, also the RNA-seq data of the ASCOT data set showed a higher coverage of exons 3 to 11 in comparison to exons 1, 1b and 2 in the “retina-eye” sample (Supplementary Fig. 3). This was not observed for other human tissues, e.g. adipose-subcutaneous or liver tissue – for these, exons 1(b) to 11 had an overall comparable coverage. This indicates that the predominant *CNGA1* transcript in the human retina only comprises exons 3 to 11.

If exons 1 and 2 are indeed absent from the predominant transcript in the human retina, *CNGA1* likely utilizes multiple TSS. The FANTOM5 project performed CAGE-seq across different human tissues and cell types to identify TSS. Transcription start sites were mapped to exon 1 and exon 1b based on *CNGA1* transcripts detected in different human tissues and cell lines (Supplementary Fig. 3). A third peak of CAGE-seq reads located to *CNGA1* exon 3 and comprised reads mainly detected in fetal eye and adult retina samples (Supplementary Fig. 3). Thus, RNA- and CAGE-seq data indicate that transcription of *CNGA1* is mainly initiated upstream of exon 3 and the predominant transcript in the human retina spans exon 3 to 11 (Supplementary Fig. 2 and 3).

### Compilation of *CNGA1* missense variants

In total, 86 *CNGA1* missense variants were identified which (1) were reported in IRD/RP-association and (2) located in functionally and structurally important channel domains (Supplementary Fig. 1; Supplementary Table 4). Of these, three *CNGA1* missense variants are novel and were identified in patients of the *CNGA1*-RP study group.

Various transcripts have been used to annotate *CNGA1* variants in the past decades and were postulated to encode CNGA1 proteins of variable length. This posed a challenge in variant acquisition and annotation. For this study, all variants were carefully (re)-annotated on the MANEselect transcript (NM_001379270.1; Supplementary Table 4), which is predicted to give rise to a 686 aa long protein (NP_001366199.1). Upstream (12 bp) of the currently defined translation initiation site (TIS) in the MANESelect transcript, an in-frame TIS is present in human *CNGA1*. This site was previously used as the original TIS for variant annotation in transcript NM_000087.3, predicted to encode a CNGA1 protein (NP_000078.2) of 690 aa.In addition, a splice isoform of *CNGA1* (NM_001142564.1) was reported and predicted to give rise to a 759 aa CNGA1 protein (NP_001136036.1). The latter, has been revised (NCBI accessed May 2025) to NM_001142564.2, now also being predicted to encode the 686 aa protein (NP_001136036.2). Due to variability in available transcripts and TIS, the same *CNGA1* variant can currently have three different annotations in patient reports, literature, or databases. To facilitate straightforward compilation and comparison with previous studies, this study provides MANESelect transcript–based annotations for the 86 *CNGA1* variants. In addition, the original published or database-reported variant descriptions are listed (Supplementary Table 4).

### Evolutionary conservation of CNGA subunits and modelling of CNGA1 missense variants in human CNGA3 channels

Initially, we evaluated the replacement of CNGA3 with CNGA1 within our aequorin-based bioassay (Solaki et al. 2023). To test this, wild-type CNGA1 was heterologously expressed in HEK293^aequo^ cells, but did not elicit detectable 8-Br-cGMP inducible luminescence responses in the bioassay – which is in contrast to wild-type CNGA3 channels (Tager et al. 2018, 2021; Solaki et al. 2023), but supported by the known difference in calcium permeability between CNGA1 and CNGA3 channels (Frings et al. 1995; Dzeja et al. 1999). Therefore, heterologous expression of CNGA1 in HEK293^aequo^ cells is not considered suitable for our bioassay and read-out.

To overcome this issue, we hypothesized that there is sufficient evolutionary conservation between human CNGA1 and CNGA3 to allow CNGA1 variants to be evaluated within the CNGA3 protein context. Alignment of CNGA1 and CNGA3 orthologues from different species indicates that the CNGA subunits of rod and cone CNG channels show high conservation especially within structurally and functionally important domains. The N- and C-terminals regions are less conserved (Supplementary Fig. 1). The human CNGA1 and CNGA3 shows an overall sequence identity of 66.5%, and 76.2% sequence conservation within structurally and functionally critical domains, rendering the CNGA3 subunit suitable to study CNGA1 variants within these conserved regions (Supplementary Table 5).

Of the 86 *CNGA1* missense variants to be studied, 71 affect aa residues that are identical in human CNGA1 and CNGA3. For these variants, the aa substitution of the IRD-associated CNGA1 variant was introduced into CNGA3 (Supplementary Fig. 1). The remaining 15 variants affect positions that are not identical between the two paralogues in humans. For these variants, the respective aa substitution observed in CNGA1 and a CNGA1/A3 homology control variant were generated.

### Evaluation of the bioassay to functionally assess *CNGA1* variants

To evaluate the functional impact of *CNGA1* missense variants, the aequorin-based assay was employed using the CNGA3 protein as a model. To validate the suitability of this assay for functional assessment of *CNGA1* missense variants and to define thresholds that differentiate functionally normal from abnormal variants, two test groups were analyzed. The control group comprised (1) three variants designated with a clinical significance of “benign” on gnomAD v4.1.0 (non-UKB) and ≥ 2 homozygous entries in the general population (Supplementary Table 6), and (2) 17 CNGA1/A3 homology control variants. Of the compiled 86 *CNGA1* missense variants, 30 variants were classified as LP prior to functional testing (Supplementary Table 4). Provided the bioassay is suitably designed, it is expected to allow discrimination between the LP and control groups based on their functional performance in the assay.

**Fig. 1:**
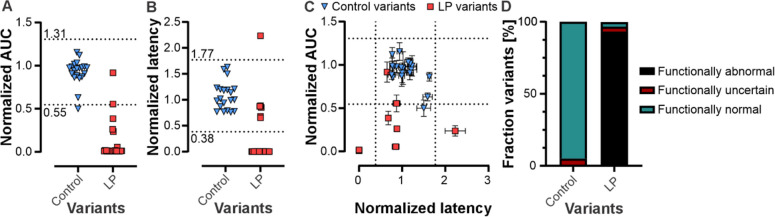
Validation of the aequorin-based luminescence bioassay to evaluate *CNGA1* variants. For validation and to establish z-score based thresholds differentiating functionally normal and abnormal variants, variants initially classified as LP (*n* = 30) and control variants (comprising three variants identified from gnomAD and having a clinical significance of benign, and 17 CNGA1/A3 homology control variants) were analyzed. Distribution of the overall normalized luminescence intensity (**A**) and the normalized latency (**B**) for the control and LP variants. **C** Scatter plot combining both parameters enabled classification of variants into functionally normal, abnormal or uncertain. **D** The assay enabled categorization of 95.0% of the control variants as “functionally normal” and 93.3% of the LP variants as “functionally abnormal”. Dotted lines in A to C represent z-score based threshold of ± 2.56xSD (*p* ≤ 0.01) based on the overall normalized luminescence intensity or the normalized latency of the control variants. At least three independent transfections were performed

In the first step, z-score-based thresholds of ± 2.56xSD corresponding to a significance level of *p* ≤ 0.01 were established from the control group for the two parameters ‘overall luminescence intensity’ (i.e., AUC) and ‘latency’. These thresholds were then applied to the normalized AUC and latency values of all control and LP variants (Fig. 1) enabling classification into functionally normal, abnormal or uncertain. Using this approach, 95% (19/20) of the variants of the control group were classified as “functionally normal” (Fig. 1). The variant p.R340H was the only control variant classified as “functionally uncertain”. In the LP group, 23 variants were identified as non-functional due to absent luminescence responses in the bioassay (Fig. 1), five variants (p.R276P, p.S316F, p.V389G, p.I392T, p.D507H) showed a reduced AUC, one variant (p.E659G) displayed an AUC within the normal range, and one variant (p.L633R) hit the threshold (Fig. 1). Of the six LP variants that showed a sufficient luminescence signal intensity in the bioassay (normalized AUC ≥ 0.02), the latency was determined. Five variants presented with a latency within the normal range and one variant (p.S316F) showed an increased normalized latency (Fig. 1). By combining the AUC and latency data, 93.3% (28/30) of the LP variants were categorized as “functionally abnormal” (Fig. 1; Supplementary Fig. 4), including five variants enabling residual channel activity (p.S316F, p.R267P, p.V389G, p.I392T, p.D507H), and 23 non-functional variants. One of the LP variants (p.E695G) was classified as “functionally normal” and one (p.L633R) as “functionally uncertain”. Since both these variants locate within the CLZ domain which is essential for homo-/heterotetrameric channel formation, the bioassay may not be suitable to assess variants within this region. For variants outside this region, the aequorin-based assay and the established statistical thresholds effectively discriminated between “functionally normal” and “functionally abnormal” *CNGA1* variants, confirming the assay's suitability for functional evaluation of missense variants in this protein. Luminescence traces of all variants are depicted in Supplementary Fig. 5 to 7.

### Functional evaluation of *CNGA1* missense variants

**Fig. 2:**
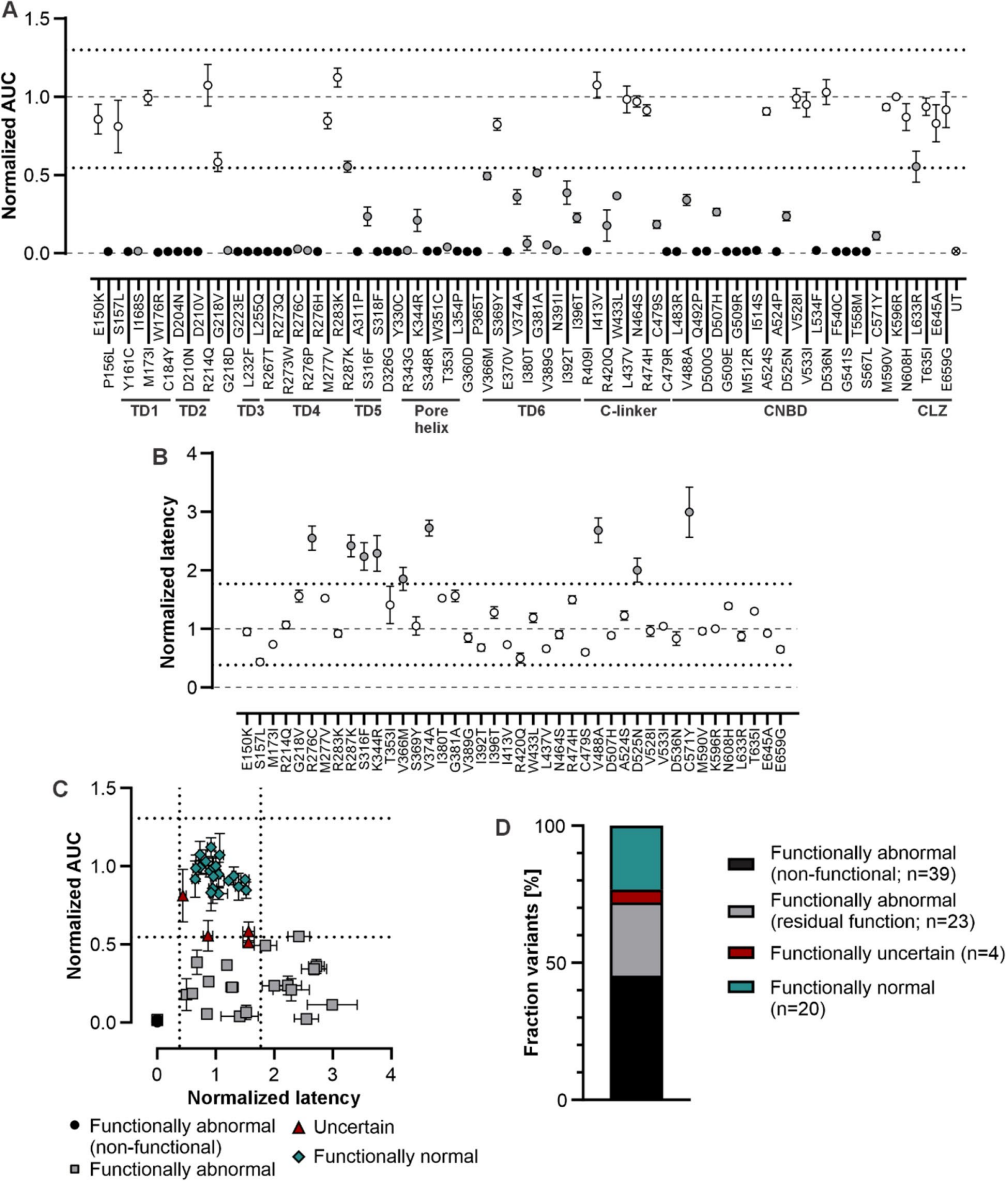
Functional evaluation of 86 *CNGA1* missense variants. **A** Presentation of the AUC normalized to CNGA3_wt_ channels and sorted by variant position. Underneath the variants, functionally and structurally important CNGA1 protein domains are indicated. Dotted lines indicate the z-score based thresholds of ± 2.56xSD. Variants whose AUC values fall within the defined thresholds appear as white circles. Variants exceeding these thresholds are depicted as grey circles when residual channel function was recorded, and as black circles when channel activity was lost. Untransfected HEK293^aequo^ cells served as controls. **B** For functional variant channels with a normalized AUC above 0.02 the latency was determined. Dotted lines indicate the z-score based thresholds of ± 2.56xSD. Variants with latency within the thresholds are depicted as white circles and variants outside the threshold as grey circles. **C** Scatter plot demonstrating the distribution of the normalized AUC and latency within the z-score thresholds (dotted lines) of ± 2.56xSD for functionally abnormal, normal or uncertain CNGA1 variant channels. **D** Functional outcome of the 86 *CNGA1* variants analyzed. For A to C, the mean ± SD of at least three independent transfections is shown. TD: transmembrane domain, CNBD: cyclic-nucleotide binding site; CLZ: C-terminal leucine zipper domain; UT: untransfected

In total, 86 *CNGA1* missense variants were functionally analyzed and evaluation of the normalized AUC indicated 21/86 (24.4%) variants being within the thresholds. Four variants (4.7%; p.G218V, p.R287K, p.G381A, p.L633R) hit the thresholds and 61 variants (70.9%) had a normalized AUC below the z-score threshold of −2.56xSD (Fig. 2; Supplementary Fig. 4). Of these 61 variants, 39 (63.9%) showed no 8-Br-cGMP inducible luminescence signal (designated as “non-functional”) and 22 (36.1%) had a reduced AUC in comparison to the control group.

Forty-two (48.8%) variants showed sufficient luminescence signal intensity enabling determination of the normalized latency (Fig. 2; Supplementary Fig. 4). Thereby, 32 variants (76.2%) depicted a normalized latency comparable to the control variant group, eight variants (19.0%) showed an increase normalized latency above the z-score based threshold of + 2.56xSD and two variants (4.8%; p.S156L, p.V366M) overlapped with the thresholds. Combining both bioassay parameters, 62 *CNGA1* variants (72.1%) were categorized as “functionally abnormal”, 20 (23.2%) variants as “functionally normal” and four (4.7%; p.S157L, p.G218V, p.G381A, p.L633R) as “functionally uncertain” (Fig. 2; Supplementary Fig. 4). Luminescence traces of all variant channels are displayed in Supplementary Figs. 5 to 7. Dot blot analysis confirmed expression of all CNGA1 variant channels and controls in transiently transfected HEK293^aequo^ cells (Supplement Fig. 8).

**Fig. 3:**
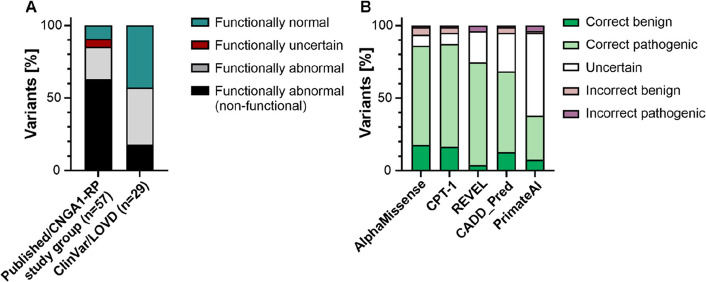
Correlation of functional data with variant origin and variant evaluation by computational predictors. **A** The *CNGA1* variants studied with the aequorin-based bioassay were identified from specialized IRD centers, literature and the public databases ClinVar and LOVD, and were sorted with respect to their functional outcome per cohort. **B** For five computational predictors, the fraction of functionally normal/abnormal variants correctly predicted as B or P as well as the fraction of incorrectly predicted variants was assessed. For the data presented in A and B, four variants locating in the CLZ of CNGA1 were not considered

To further assess the performance of the bioassay, functional classifications of *CNGA1* variants were analyzed according to their source (Fig. 3). Variants derived from specialized IRD centers or published IRD cohort studies are typically linked to IRD or RP phenotypes, whereas ClinVar-derived variant entries may lack consistent phenotypic association due to their broader and less curated nature. We hypothesized that the former are more likely to be associated with disrupted CNG channel function and, therefore, should more frequently be classified as "functionally abnormal". As expected, 85.2% of published/*CNGA1*-RP study group variants showed to be “functionally abnormal” using the aequorin-based bioassay. In contrast, variants retrieved from public databases without direct phenotype association showed a lower proportion of functionally abnormal variants (57.1%; Fig. 3).

As computational tools are commonly used to assess missense variant effects in silico, the correlation of our functional data with the prediction of five selected VEPs was assessed. These comprised four commonly used VEPs and one recently developed VEP (CPT-1), for which data from deep mutational scanning approaches were included in the training to enhance prediction accuracy. Therefore, the normalized AUC and latency were combined into a functional score for each of the 82 studied *CNGA1* missense variants (outside the CLZ domain) and correlated with the VEP scores (Supplementary Fig. 9). A high correlation of the functional data from our bioassay and AlphaMissense (ρ = −0.68; *p* ≤ 0.0001) and CPT-1 scores was observed (ρ = −0.64; *p* ≤ 0.0001) whereas a moderate correlation was detected with REVEL (ρ = −0.55; *p* ≤ 0.0001) and CADD_Phred (ρ = 0.−44; *p* ≤ 0.0001) scores (Supplementary Fig. 9). Based on the VEP score, variant effects can be categorized as B, uncertain or P applying thresholds as suggested by Jagota and colleagues, Pejaver and coworkers (Jagota et al. 2023; Pejaver et al. 2022), https://alphamissense.hegelab.org, or as determined via ROC analysis for CPT-1 (Supplementary Fig. 9). AlphaMissense and CPT-1 correctly predicted the effect of 86.1% and 87.3% of functionally normal or abnormal variants as B or P, respectively (Fig. 3). The performance of the VEPs REVEL and CADD_Phred was slightly less reliable with correctly predicting the effect of 74.7% and 68.4% of functionally normal or abnormal variants, respectively. Discordant prediction rates for these four VEPs ranged from 3.8% to 6.3% (Fig. 3B). The correlation of the *CNGA1* variant functional scores and the PrimateAI scores was very low (ρ = −0.17; *p* = 0.1261), as were the proportion of correctly classified functionally normal or abnormal variants as B or P (38.0%) indicating that this VEP is not suitable for predicting *CNGA1* missense variant effects (Fig. 3; Supplementary Fig. 9).

### Minigene splice assay

For the two exonic *CNGA1* variants c.448G > A/p.E150K and c.653G > T/p.G218V, the VEP SpliceAI predicted an effect onto splicing with scores > 0.1 (Supplementary Table 7). Therefore, both variants were studied via in vitro minigene splice assays. For variant c.448G > A, the fraction of canonically spliced transcripts was reduced (60.8%), whereas the number of spliced transcripts in which *CNGA1* exon 9 (28%) or 9/10 (11.3%) were skipped was increased (Supplementary Fig. 10). For the wild-type CNGA1_ex7-10 minigene, only 1% of spliced transcripts presented with exon 9 skipping and 99% represented canonically spliced transcripts. Exon 9/10 skipping was only detected after sub-cloning of RT-PCR products and was not visible on the agarose gel image. Skipping of exon 9 is in-frame and predicted to result in the deletion of 36 aa within TD1 whereas skipping of exons 9/10 is expected to result in a frameshift and premature termination codon upon translation (Supplementary Table 7). For variant c.653G > T canonical splicing was detected, but transcripts were also identified in which various cryptic acceptor sites within exon 11 were used (Supplementary Fig. 11). The latter resulted in the deletion of the first 194 bp to 1071 bp of exon 11. These misspliced transcripts are predicted to result in frameshifts and premature termination codons when translated, giving rise to CNGA1 proteins that lack most functionally and structurally relevant domains (Supplementary Table 7). Thus, for the variants c.448G > A/p.E150K and c.653G > T/p.G218V incomplete missplicing could be confirmed.

### Application of the functional data on the ACMG/AMP classification of *CNGA1* variants

**Fig. 4:**
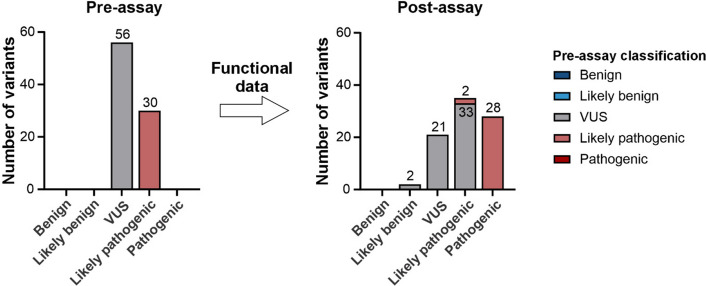
ACMG/AMP-based classification of *CNGA1* missense variants prior and post application of the functional data. Application of functional data enabled re-classification of 62.5% of VUS and confirmed disease-relevance for 93.3% of LP variants

To evaluate whether and how the functional data from the aequorin-based bioassay and in vitro minigene assays influence variant classification, the 86 *CNGA1* missense variants were classified for their disease relevance according to ACMG/AMP criteria. Initially, 56 variants (65.1%) were categorized as VUS and 30 (34.9%) as LP (Fig. 4). Using functional data from the presumed P and B control variants, OddsPath scores of 18.7 (P variants) and 0.07 (B variants) were calculated (Supplementary Table 8), enabling application of PS3 for functionally abnormal variants and BS3_supporting for functionally normal variants. For variants that were “functionally uncertain” (p.S157L, p.G218V, p.G381A, p.L633R) and/or located within the CLZ domain (p.L633R, p.T6315I, p.E645A, p.E659G), neither BS3 nor PS3 were applied. Following the recommendations of Walker and colleagues, the in vitro minigene splice results for the variants c.448G > A/p.E150K and c.653G > T/p.G218V were not considered for ACMG/AMP classification as both variants showed incomplete missplicing (Walker et al. 2023). Incorporation of the functional data enabled re-classification of 73.3% of variants. Of the 30 CNGA1 variants initially classified as LP, 28 variants (93.3%) could be re-categorized as P thereby confirming their disease relevance. Two variants (6.7%; p.E659G and p.L633R) remained as LP (Fig. 4). Of the 56 VUS, 33 variants (58.9%) could be re-classified as LP, and two variants (3.6%; p.V528I, p.N608H) as LB (Fig. 4). The classification of 15 VUS (26.8%) remained unaltered despite application of BS3 (14 variants) or PS3 (one variant). Overall, 62.5% (35/56) of the variants initially classified as VUS could be re-reclassified (Fig. 4).

## Discussion

This study aimed to generate functional data to support the assessment of missense variants in *CNGA1* for their clinical relevance in IRD/RP patients. Functional data are urgently needed, as 65.1% of IRD-associated *CNGA1* missense variants were initially classified as VUS, and a clinical trial targeting *CNGA1*-RP is currently ongoing (NCT06291935).

During the compilation of *CNGA1* missense variants from literature and public databases, variability in variant annotation was observed. These inconsistencies arise from the use of different transcripts in genomic databases and multiple transcript revisions over time, including recurrent changes to the annotated TIS. RNA-seq data from human post-mortem retinal tissue and CAGE-seq data from various human tissues and cell lines indicated that transcription of *CNGA1* in the adult retina initiates upstream of exon 3 (as numbered in NM_001379270.1), and that the predominant retinal transcript includes only exons 3 to 11. Additionally, exon 3b, which is part of transcript NM_001142564, was detected only at low levels in RNA-seq data from post-mortem human retinal tissue. The findings suggest that *CNGA1* utilizes at least three distinct TSS across different tissues. Since the TIS locates within exon 4, these alternative TSS result in transcripts with variable 5′UTRs, potentially affecting mRNA stability and/or translation efficiency (Mignone et al. 2002; Wang et al. 2005).

*CNGA1* variants have been reported in IRD patients across diverse populations, and earlier studies suggested a higher prevalence in Asians compared to Europeans (Katagiri et al. 2014). However, this assumption is not supported by recent larger cohort studies. Literature research on IRD cohort studies from different populations showed that within genetically solved IRD cohorts, *CNGA1* variants have a prevalence ranging from 0 to 1.7%; the highest prevalences of > 1% being observed in Japanese, Chinese, Turkish and Mexican cohorts (Supplementary Table 9). In clinically well-defined cohorts, the frequency of *CNGA1* variants was higher, reaching approximately 5% in genetically resolved patients with autosomal recessive RP. This was observed across cohorts from Japan, Germany, Spain, and Mexico, suggesting that *CNGA1* variants are not more prevalent in specific populations (Supplementary Table 9) (Eisenberger et al. 2013; Katagiri et al. 2014; Villanueva-Mendoza et al. 2021; Perea-Romero et al. 2021).

To assess the functional impact of *CNGA1* missense variants, we attempted to adapt our aequorin-based bioassay by replacing CNGA3 with CNGA1, which may be considered the major limitation of our study. (Tager et al. 2018, 2021; Kohl et al. 2021; Solaki et al. 2023). However, neither homomeric (CNGA1 alone) nor heteromeric (CNGA1 + CNGB1) rod CNG channels heterologously expressed in HEK293^aequo^ cells generated ligand-induced luminescence responses. This aligns with previous findings using the calcium-sensitive fluorescent dye Rhod-4AM, which detected calcium fluxes for CNGA3 and CNGA2 but not for CNGA1 channels expressed in HEK293 cells (Jacobson et al. 2019). In contrast, Pavlou and colleagues reported successful detection of CNGA1-mediated calcium fluxes using the genetically encoded calcium indicator GCaMP6s in transduced HeLa cells (Pavlou et al. 2022). This discrepancy likely reflects the lower calcium permeability of rod compared to cone CNG channels (Frings et al. 1995; Dzeja et al. 1999). Therefore, calcium indicators with lower sensitivity, such as Rhod-4AM and aequorin, may be insufficient to detect the relatively small CNGA1-mediated calcium currents, whereas the higher-sensitivity GCaMP6s may be able to do so. Reliable assessment of mutant CNG channel function requires kinetic measurements of calcium flux, but prior work reported only absolute fluorescence changes, limiting insight into channel activity dynamics. Notably, CNGA1 function has been successfully characterized in HEK293 cells using patch-clamp recordings based on sodium currents, further suggesting that limited calcium conductivity is the main constraint. (Dryja et al. 1995; Xue et al. 2022). Further attempts to optimize the bioassay (e.g. variable calcium concentrations in the CI buffer, extended measurement and photon collection times or usage of coelenterazine derivatives) enabling quantification of CNGA1 channel mediated calcium fluxes were not successful (data not shown).

To circumvent this issue, we leveraged the high evolutionary conservation of CNGA paralogues and used CNGA3 as a surrogate to study CNGA1 variants. Validation with B and LP CNGA1 variants confirmed suitability of the bioassay for variants outside the CLZ domain. For assay evaluation, three missense variants were selected based on their clinical significance as B on gnomAD v4.1.0 and the presence of at least two homozygous individuals in the general population. The common CNGA1 polymorphism p.D114N was excluded because it lacked a homologous position in human CNGA3. Whereas two control variants were correctly classified as functionally normal, the B control variant p.R340H was classified as “functionally uncertain” with the bioassay. This variant affects an evolutionary conserved aa located close to the pore helix and has a grpmax filtering AF of 0.01 in the general population. It has been reported homozygously in 19 individuals from South Asia. The p.R340H variant may represent a hypomorphic allele, or the functional changes detected in the bioassay may not be biologically relevant in photoreceptors. In rod and cone outer segments, only a small fraction of CNG channels is active under physiological conditions due to low intracellular cGMP levels, and rod channel activity is further modulated by calmodulin (Chen et al. 1994; (Kolb et al., 1995). Therefore, some degree of functional variability caused by missense variants may be tolerated in vivo.

As an initial validation step of the aequorin-based bioassay, we tested the 30 *CNGA1* variants ACMG/AMP classified as LP prior to functional data application and confirmed that our approach successfully identified 93.3% (28/30) as “functionally abnormal”. Two LP variants need to be highlighted: p.E659G appeared “functionally normal”, while p.L633R was “functionally uncertain”. Both variants locate to the CLZ domain, which is only present in CNGA but absent in CNGB subunits. This domain mediates an initial trimeric interaction of CNGA subunits and a preferential recruitment of a CNGB subunit to form the tetrameric channel (Trudeau & Zagotta 2002; Zhong et al. 2003; Shuart et al. 2011). Thus, the aequorin-based bioassay may not be suitable to reliably evaluate missense variants within the CLZ, which represents another limitation of our study.

In total, 23.3% *CNGA1* variants were classified as “functionally normal” and the majority of variants (72.1%) as “functionally abnormal” using the aequorin-based bioassay. A high fraction (62.9%) of functionally abnormal variants did not show (reproducible) ligand-inducible calcium responses in the bioassay. This is in line with previous studies showing that missense variants frequently severely impair CNG channel trafficking and function, as observed in the absence of signals in calcium imaging and electrophysiological studies (Dryja et al. 1995; Muraki-Oda et al. 2007; Reuter et al. 2008; Koeppen et al. 2008, 2010; Solaki et al. 2023).

Up to now, functional data were only available for a very limited number of *CNGA1* variants, e.g. the common RP-associated variant p.S316F which was categorized as “functionally abnormal” and showed residual channel function in the bioassay. This finding is consistent with an earlier report demonstrating that CNGA1_S316F_ channels retain functionality (Dryja et al. 1995), however ligand-induced responses were observed in fewer inside-out patches compared to wild-type channels heterologously expressed in HEK293 cells, suggesting that the variant impairs CNG channel surface expression (Dryja et al. 1995). The p.D204N variant has previously been shown to reduce the total mutant CNGA1 protein amounts as well as the surface integration of mutant channels when heterologously expressed in HEK293 cells (Gao et al. 2019). Accordingly, the variant p.D204N, which was classified as LP by the ACMG/AMP criteria, proved to be “functionally abnormal” in the bioassay. The two variants, p.V374A and p.G381A, initially classified as VUS, were previously analyzed via alanine-scanning mutagenesis using heterologous expression in *Xenopus laevis* oocytes. Both variants supported channel function but converted the normally voltage-insensitive homomeric CNGA1 channel voltage-sensitive. It was hypothesized that under physiological voltages, these mutant channels would remain predominantly closed even at saturating cGMP levels (Martinez-Francois et al. 2009). Consistent with this, CNGA1_V374A_ channels showed altered normalized latency and AUC in the bioassay, leading to their classification as “functionally abnormal”. In contrast, CNGA1_G381A_ channels exhibited a modest reduction in overall luminescence signal intensity, resulting in a classification of “functionally uncertain”. The variants p.F540C, p.G541S, and p.T558M alter aa residues critical for cGMP binding, as demonstrated by cryo-electron microscopical structural analysis and functional studies (Xue et al. 2022; Pliushcheuskaya et al. 2024). Accordingly, channels harboring any of these variants were non-functional in the luminescence bioassay.

Fifty-four *CNGA1* variants (excluding three variants in the CLZ) were identified likely or confirmed biallelic in patients with clinically diagnosed IRD or RP, suggesting a high likelihood of disease-association. Indeed, 85.2% of these variants were “functionally abnormal” in the bioassay, supporting their disease relevance. Conversely, only 9.3% (5/54; p.E150K; p.R214Q, p.R283K, p.S369Y, p.N608H) were “functionally normal”. The variant p.N608H was reported to not segregate with the disease (Gonzalez-del Pozo et al. 2011). Thus, the functional data support that this variant is a rare B variant and is not associated with IRD or RP in the patient. The variants p.R214Q and p.S369Y were identified in a Japanese RP cohort and have been reported as VUS. Unfortunately, more detailed information about patient genotypes and IRD phenotypes was not available (Koyanagi et al. 2019). The missense variants p.R214Q and p.S369Y had inconsistent in silico evaluations with one or two out of four VEPs (not considering PrimateAI) suggesting a B effect and only REVEL predicting a P effect. Thus, the “functionally normal” evaluation in the bioassay strongly supports that these *CNGA1* variants are not IRD/RP associated. The variant p.R283K was identified in Japanese RP patients (Katagiri et al. 2014; Koyanagi et al. 2019). Katagiri and colleagues classified this variant as not disease-causing as it was also detected in unaffected Japanese controls. This conclusion is supported by our functional data. The variant p.E150K – also reported in a Japanese cohort – was predicted B by the five VEPs assessing aa substitutions, but was indicated as potentially spliceogenic by SpliceAI, albeit with a low score (Koyanagi et al. 2019). Accordingly, this variant did not impair channel function as observed in the bioassay, but impaired splicing in in vitro minigene splice assays. The disease relevance of the p.E150K is not conclusively clarified, as still a high fraction of correctly spliced transcripts was observed in the in vitro splice assay. Nevertheless, if this variant is indeed causative for IRD/RP, it will rather exert its pathogenic effect as a splice variant than as a missense variant.

The strong concordance between the functional data obtained from the aequorin-based bioassay, previous functional studies on *CNGA1* variants, and molecular genetic findings from RP/IRD patients confirms the reliability of the bioassay and supports the validity of using CNGA3 as a surrogate for functional evaluation of CNGA1 missense variants.

Within the published variants, 5.6% (3/54; p.S157L, p.G218V and p.G381A) were identified as “functionally uncertain” in the bioassay. Thus, the disease relevance for the variants p.S157L and G381V remains elusive. For the variant c.653G > T/p.G218V, 4/4 VEPs (not considering PrimateAI) suggested a pathogenic effect for the aa substitution and SpliceAI indicated an effect on splicing at the nucleotide level. The latter is supported by the in vitro minigene splice assay, which demonstrated incomplete missplicing caused by the variant. Thus, the c.653G > T/p.G218V is expected to be disease-relevant and may act via two mechanisms: as a spliceogenic variant generating transcripts that would encode proteins lacking most functionally important domains, or, if correctly spliced, producing mutant CNGA1 subunits with impaired function (Supplementary Table 4 and 8).

The most common pathogenic *CNGA1* variant is p.S316F with a grpmax filtering AF of 0.002 in the general population. Notably, two variants (p.R420Q and p.K344R) with higher AF in the general population were found to impaired CNG channel function. The p.R420Q variant (grpmax AF = 0.0079) is common in Asian populations, and p.K344R (grpmax AF = 0.0056) is prevalent in African/African-American populations based on gnomAD v4.1.0 (non-UKB). The p.R420Q variant has been reported in Japanese RP cohorts (Katagiri et al. 2014; Koyanagi et al. 2019), but was classified as non-pathogenic due to its relatively high frequency in unaffected individuals including two homozygous individuals (0.43% in Japanese controls). However, in our bioassay, CNGA3_K344R_ produced a luminescence response nearly identical to that of CNGA3_S316F_ channels, and CNGA3_R420Q_ channels also showed a markedly reduced response, indicating severely impaired channel function (Supplementary Fig. 4). These findings suggest that p.R420Q and p.K344R may represent disease-associated alleles. *CNGA1*-associated RP is characterized by early-onset night blindness but typically progresses slowly. This could potentially contribute to the observation of homozygous *CNGA1* variant carriers in gnomAD (Gerhardt et al. 2023; Colombo et al. 2024).

VEPs are routinely used in medical genetics to assess missense variants. The reliability of five VEPs in assessing *CNGA1* missense variants was evaluated. The VEPs were selected based on the variety of information they are trained on, as this can affect their reliability: REVEL and CADD_Phred combine scores from multiple tools, integrating evolutionary conservation, functional annotation, and allele frequency data; REVEL explicitly incorporates allele frequencies, while CADD_Phred is scaled to all potential single nucleotide variants (SNVs) in the human genome (Ioannidis et al. 2016; Rentzsch et al. 2019). AlphaMissense primarily leverages protein sequence and structural information (Cheng et al. 2023). PrimateAI relies on evolutionary protein conservation in primates (Gao et al. 2023). CPT-1 integrates evolutionary sequence conservation, protein structural data, and was trained on deep mutational scanning (DMS) datasets (Jagota et al. 2023). A recent evaluation of VEP performance against DMS data from 36 proteins revealed substantial variability among predictors, with average correlations ranging from ρ = 0.3 to 0.8; the top performers were CPT-1 and AlphaMissense (Livesey & Marsh 2025). Consistent with this, our functional data showed high correlation with AlphaMissense and CPT-1 scores, whereas REVEL and CADD_Phred correlated moderately well. PrimateAI proved inappropriate for *CNGA1* missense variant evaluation. The four good performing VEPs shared a critical limitation: misclassification of variants, either labeling functionally abnormal variants as B or functionally normal variants as P. Notably, variant p.L437V was incorrectly predicted as P by 2/4 VEPs, and p.K344R, p.I380T and R287K were misclassified as B by 2–3/4 VEPs, respectively. Other misclassifications varied between predictors. These inconsistencies and contradictory classifications represent a major challenge for the in silico interpretation of variants for their clinical relevance. Functional data is therefore key to overcoming these issues.

Of the 86 *CNGA1* variants studied, the majority (65.1%) were initially classified as VUS following the ACMG/AMP recommendations. Considering our functional data for variant classification, 62.5% of the VUS could be re-classified to LB (n = 2) and LP (n = 33). Thus, the disease relevance of these variants could be refuted or confirmed which represents the major outcome of this study. Of the 30 initially LP classified variants, a pathogenic effect could be confirmed for 93.3%. For 15 of the VUS the classification was not changed after applying the functional score: the functionally abnormal variant p.L344R remained a VUS due to its high AF in the general population. For 14 functionally normal VUS the application of the criterion BS3 had no effect on variant classification, as these were rare variants (PM2). In addition, three variants were suggested P by REVEL (PP3).

As already indicated, our study has limitations: i., the assay used CNGA3 as a surrogate, and variants were not directly assessed in CNGA1 channels. However, using known LP *CNGA1* variants, we demonstrated that this strategy was reliable for studying variants in the TDs, pore, C-linker and CNBD, but not for variants in the CLZ. For variants in this domain, other methods, such as co-immunoprecipitation, may be better suited to determining their clinical significance. ii., native rod and cone CNG channels are heterotetrameric (CNGA + CNGB), whereas the variants were assessed in homomeric channels (CNGA only). Previous studies on cone CNG channels showed that the modulatory CNGB3 subunit can partially rescue the function of mutant CNGA3 subunits in heterotetrameric channels; nonetheless, this rescue was incomplete, and mutant heterotetrameric channels did not fully regain wild-type like characteristics (Reuter et al. 2008; Koeppen et al. 2008), indicating that functional evaluation of main subunit (CNGA) variants in homomeric channels is a reliable approach. iii., CNGA1 and CNGA3 differ in biophysiological properties, e.g. calcium permeability and gating, which may not be fully reflected in our experimental setup. iv., mutant CNG channels were heterologously expressed in HEK293 cells, which lack the complex morphology and physiology of photoreceptors, including specialized compartments like the outer segment and transport along the cilium. While HEK293 cells are appropriate for functional evaluation and assessing overall plasma membrane targeting of CNG channels, they do not permit evaluation of targeting to specific cellular compartments. Nevertheless, it has been demonstrated that outer segment targeting of the rod CNG channel is mediated by the CNGB1 subunit rather than the CNGA1 subunit (Kizhatil et al. 2009; Pearring et al. 2021).

In conclusion, the aequorin-based bioassay enabled functional evaluation of *CNGA1* missense variants, thereby significantly advancing our understanding of their disease relevance and enhancing molecular genetic diagnostics in patients.

## Supplementary Information

Supplementary Material 1.

Supplementary Material 2.

## Data Availability

The transcriptomics data supporting the findings of this study are openly available in ArrayExpress via the accession number E-MTAB-4377. Additional data used and/or analysed during the current study are available from the corresponding author upon reasonable request.

## Abbreviations

aa: Amino acid
ACMG: American College of Medical Genetics and Genomics
AF: Allele frequency
AMP: Association of Molecular Pathology
ASCOT: Alternative splicing catalog of the transcriptome
AUC: Area under the curve
B: Benign
CAGE-seq: Cap analysis of gene expression sequencing
cDNA: Complementary DNA
CLZ: C-terminal leucine zipper
CNBD: Cyclic nucleotide-binding domain
CNG: Cyclic nucleotide-gated
GTEx: Genotype-Tissue Expression
HEK293 cells: Human embryonic kidney 293 cells
HGMD: Human Gene Mutation Database
IRD: Inherited retinal dystrophy
IVM: *in vitro* Mutagenesis
LB: Likely benign
LOVD: Leiden Open Variation Database
LP: Likely pathogenic
NCBI: National Center for Biotechnology Information
P: Pathogenic
RP: Retinitis pigmentosa
RT: Reverse transcription
SD: Standard deviation
TD: Transmembrane domain
TIS: Translation initiation site
TSS: Transcription start sites
UCSC: University of California Santa Cruz
UTR: Untranslated region
VEP: Variant effect predictor
VUS: Variant of uncertain significance
