# Supplementary material for: Systematic functional evaluation of CNGA1 missense variants associated with retinitis pigmentosa
Source: Mol Med. 2026 May 27;32:80. doi: 10.1186/s10020-026-01518-0 (PMC13214374; doi:10.1186/s10020-026-01518-0)
Supplement: Supplementary file 1 — Supplementary Material 1. [file 10020_2026_1518_MOESM1_ESM.docx]

**Systematic functional evaluation of *CNGA1* missense variants associated with retinitis pigmentosa**

Peggy Reuter^1^, Jennifer Schroeder^1^, Marc Sturm^2^, Mathieu Quinodoz^3^, Veronika Vaclavik^4^, Miriam Bauwens^5^, Marieke De Bruyne^5^, Bart Leroy^6^, Joseph van Aerschot^7^, Katarina Stingl^8^; Susanne Kohl^1^

**
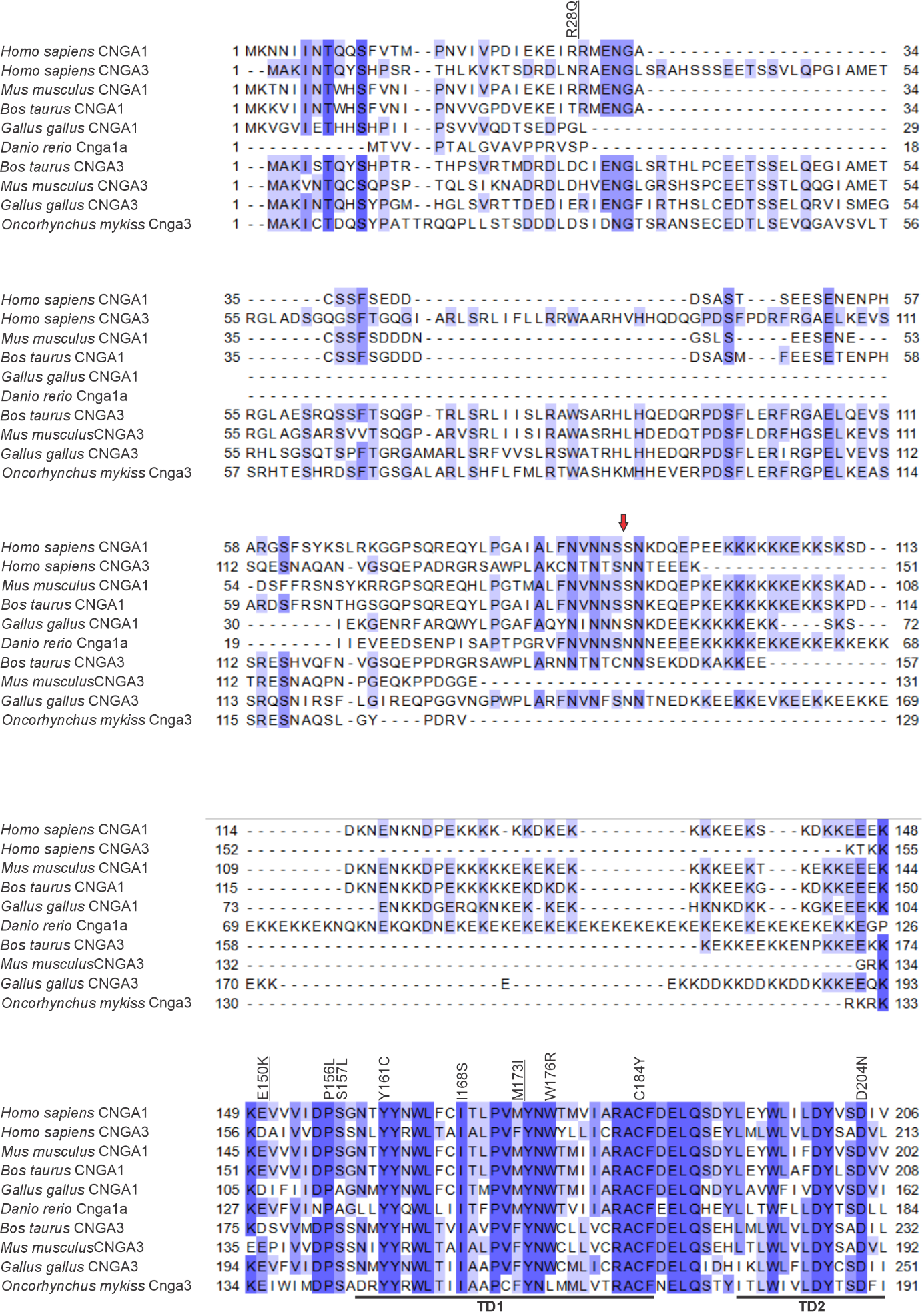

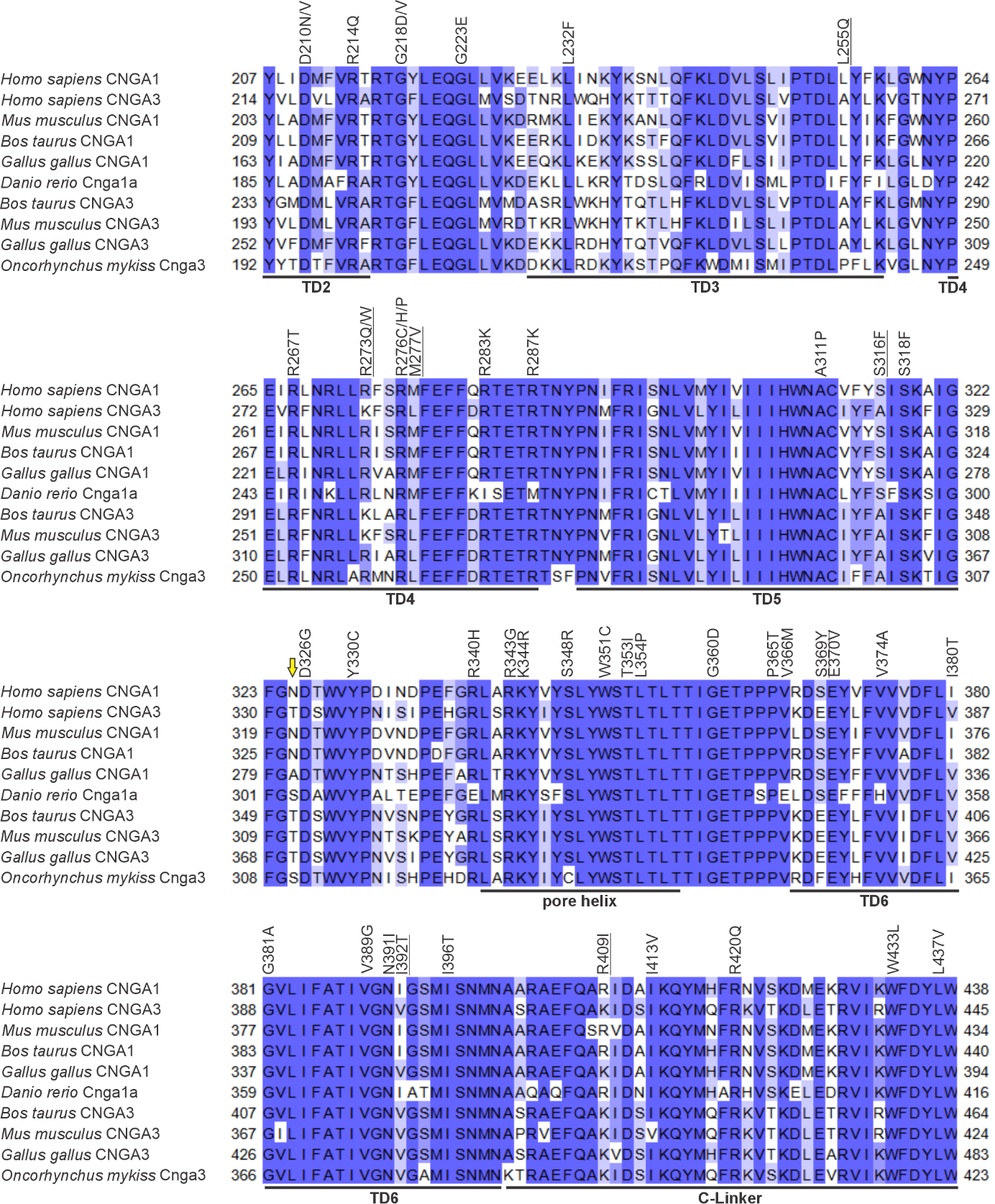
**

**
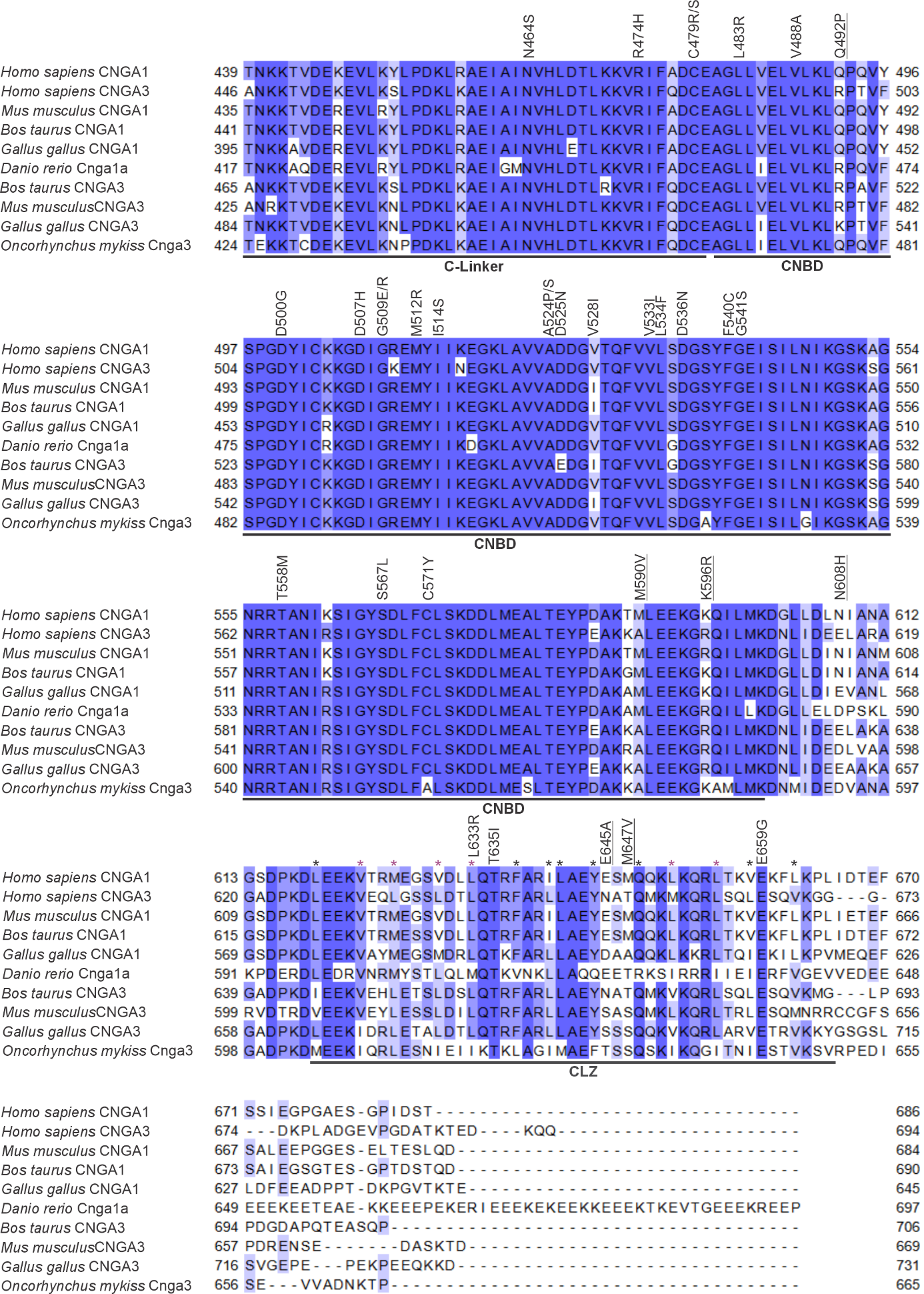
**

Supplementary figure 1: Protein alignment of CNGA1 and CNGA3 orthologues from different species. Protein alignment demonstrates high conservation within functionally and structurally important domains. Amino acid residues are highlighted in blue based on sequence identity with a threshold ≥50%. The CNGA1 variants analyzed in this study are given above the alignment, and variants, for which CNGA1/A3 homology control variants were also investigated, are underlined. Functionally and structurally important domains are indicated below the alignments and are based on (Shuart et al., 2011; Xue et al., 2022) and the AlphaFold prediction for human CNGA1 (AF-P29973-F1-v4). The red arrow indicates the suggested aminoterminal cleavage site between the two adjacent serine residues described in bovine CNGA1 (Molday et al., 1991). The yellow arrow labels an N-glycosylation site within a conserved N-X-S/T motif in the linker between TD5 and the pore helix (Rho et al., 2000). Asterisks within the CLZ domain indicate the hydrophobic residues of the heptad repeats with purple asterisk indicating residues essential for trimeric, homotypic interactions between CNGA subunits (Shuart et al., 2011; Zhong et al., 2003; Zhong et al., 2002). Percentages of amino acid sequence identity of the CNGA paralogues and orthologues are given in Supplementary table 5. NCBI reference numbers of the CNGA1 and CNGA3 protein sequences used to establish the alignment are provided in Supplementary table 1. TD: transmembrane domain; CNBD: cyclic nucleotide-binding domain; CLZ: C-terminal leucine zipper domain.


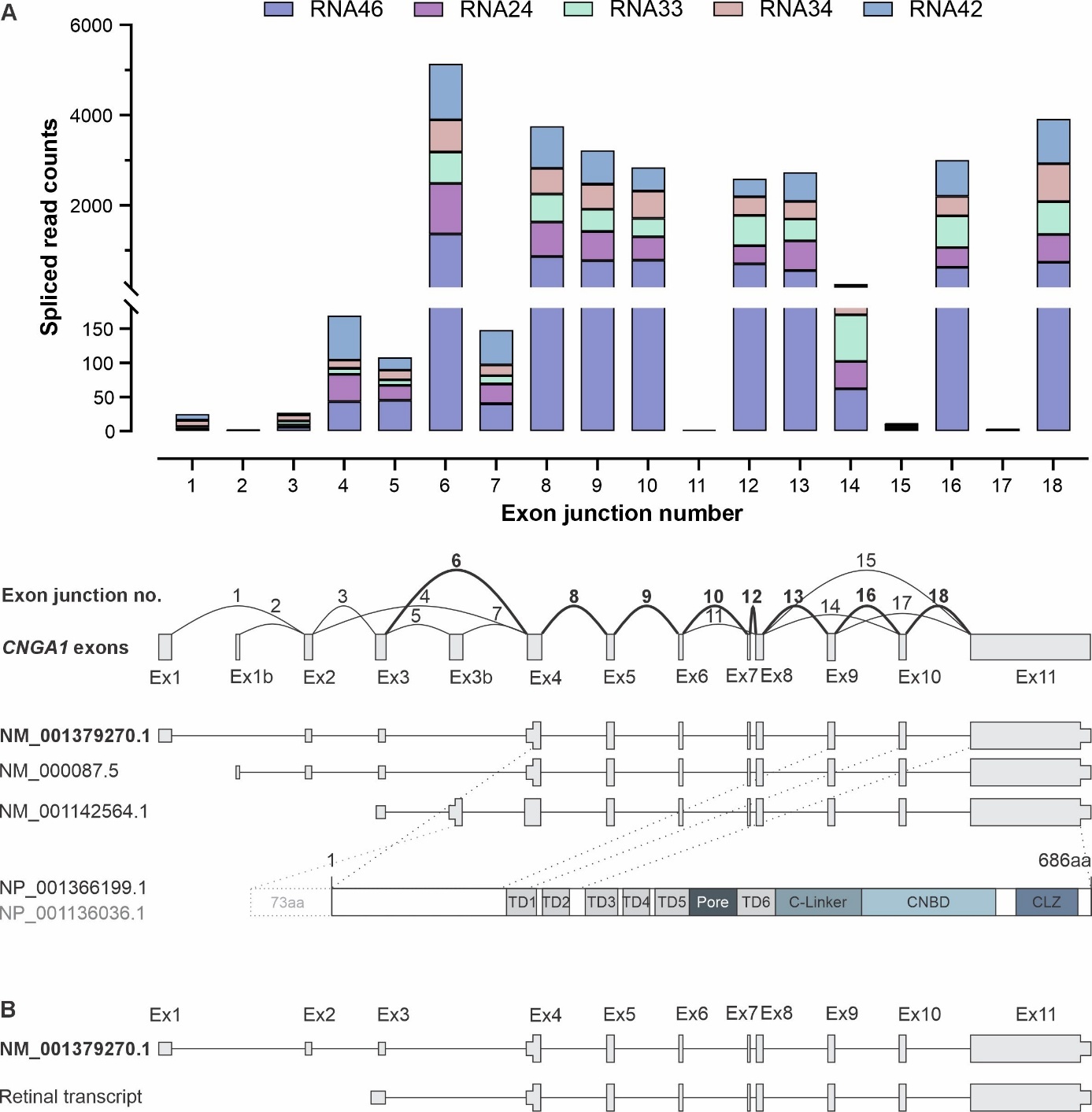


Supplementary figure 2: Overview of *CNGA1* transcript splice isoforms observed in RNA-seq data of post-mortem human retina samples. (A) In the graph (upper panel), the spliced read counts per *CNGA1* exon junction (numbers 1 to 18) are presented for four distinct post-mortem human retina samples from the ArrayExpress dataset E-MTAB-4377 (Pinelli et al., 2016). In the middle panel, the identity of the exon junctions is illustrated in relation to the *CNGA1* exons. Exon junctions with a total ≥1000 spliced reads are depicted in bold. In the retinal samples, the spliced read counts for exons upstream of exon 3 are lower in comparison to exon 3 to exon 11 spliced read counts. A relevant fraction (>250 spliced reads) of alternative splicing was observed for exon 9 (junction 14). Other junctions which are indicative of alternative splicing (junctions 4, 5 and 7) show a total number of spliced reads ≤150. Currently, three different *CNGA1* transcripts (lower panel) have been used for variant description in literature and public databases, mainly varying in the exons upstream of exon 4 of *CNGA1*. The MANEselect transcript NM_001379270.1 is indicated in bold. The transcripts NM_001379270.1 and NM_000087.5 are predicted to encode for a 686 aa protein. The transcript NM_001142564.1 includes the alternative exon 3b, which contains an alternative translation initiation codon and thus was initially predicted to encode for a 759 aa protein. Of note, NM_001142564.1 has recently been updated to NM_001142564.2 and is now also predicted to have the translation initiation side in exon 4 and to encode for the 686 aa CNGA1 protein. (B) Structure of the predominant *CNGA1* transcript in adult human retina in comparison to the MANEselect transcript (bold).


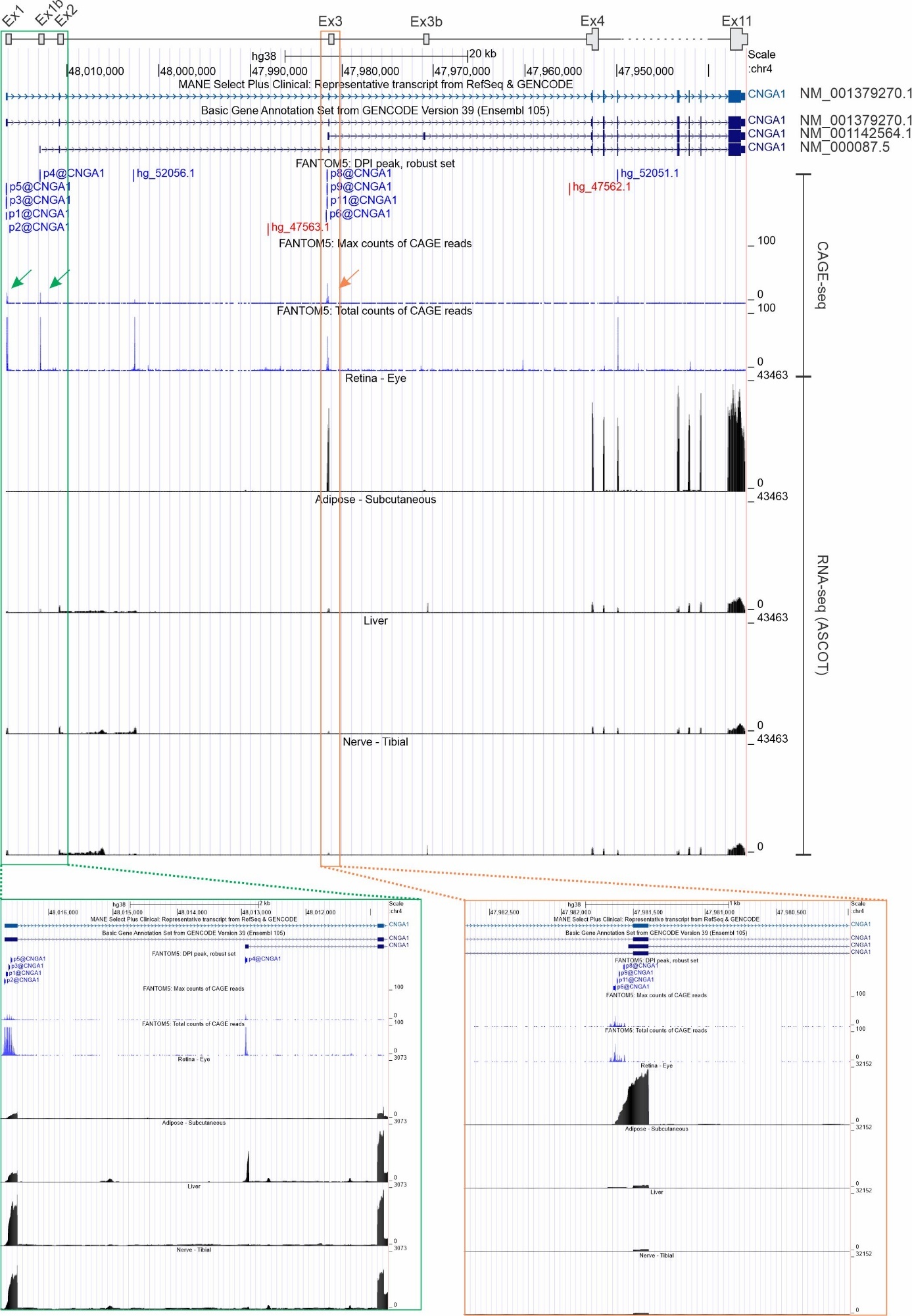


Supplementary figure 3: CAGE-seq and ASCOT RNA-seq data reveal an alternative transcription start site for the retinal *CNGA1* transcript, predominantly comprising only exons 3 to 11 of the MANEselect transcript. The ASCOT data set was group-auto scaled to the retinal sample. *CNGA1* exhibited strong expression in the “retina-eye” dataset and only low expression in other tissues, including adipose tissue, liver, and nerve, which were selected as representative non-retinal samples. RNA-seq reads cover all 11 exons of *CNGA1* (exon nomenclature adapted from Supplementary figure 1) in adipose, liver and nerve samples. Exons 1 and 2 showed low coverage in the “retina-eye” sample, whereas exons 3 to 11 are highly covered. CAGE-seq reads indicative of transcription start sites peak at exons 1 and 1b (green arrows) and at exon 3 (green arrow). CAGE-seq peaks p1-, p2-, p3-, p4- and p5@CNGA1 originated primarily from diverse types of tissues and cells studied within the FANTOM5 project, with fetal eye samples showing low signals at p1-, p4- and p2@CNGA1. In contrast, CAGE-seq peaks p6-, p8-, p9- and p11@CNGA1 predominantly arise from fetal eye and adult retina samples. These data strongly suggest that an alternative transcription start site upstream of exon 3 of *CNGA1* gives rise to a transcript composed of exons 3 to 11, predominantly expressed in the adult human retina.


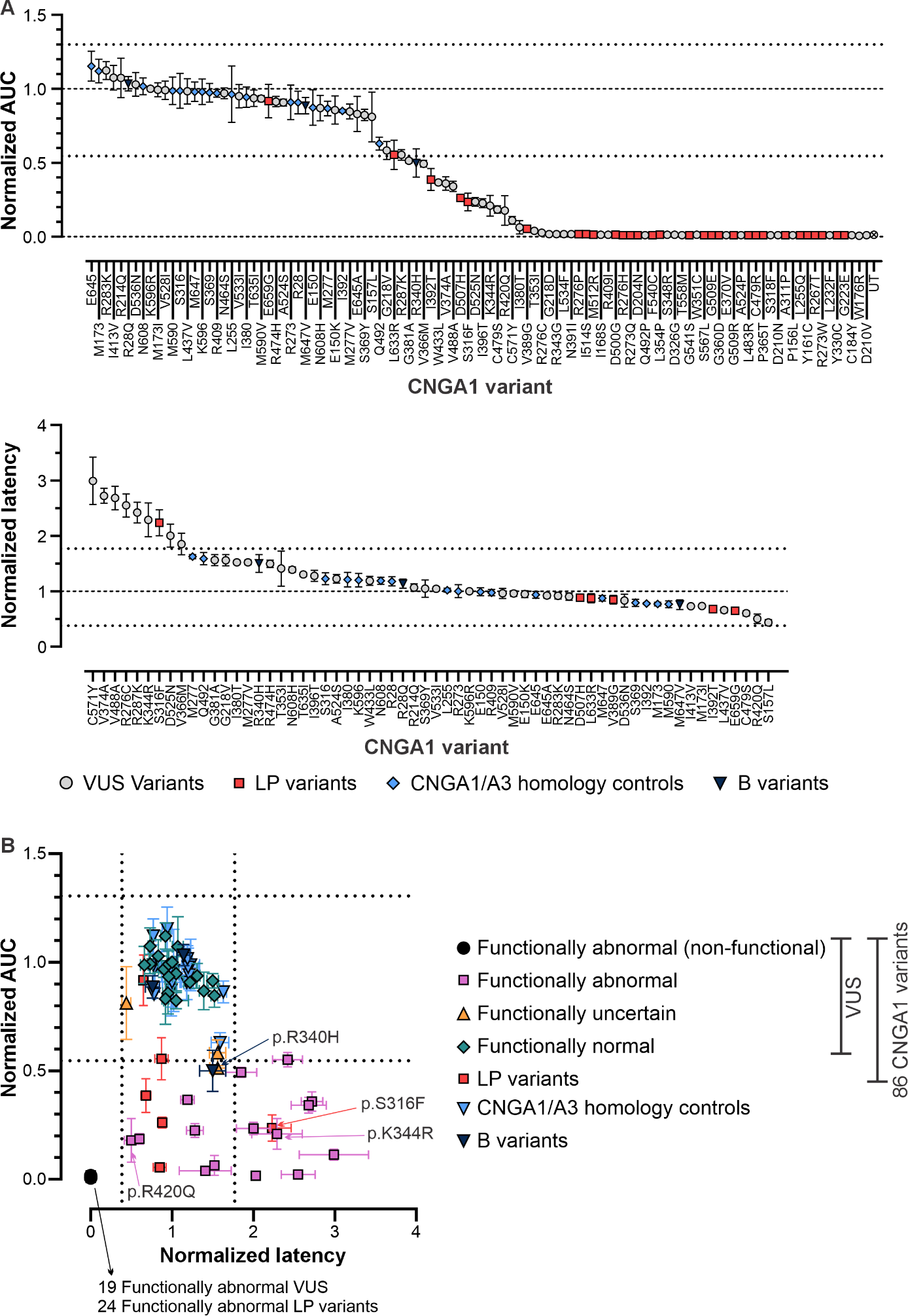


Supplementary figure 4: Functional bioassay data of the CNGA1 variants in comparison to controls. (A) Descending normalized AUC (upper panel) for variants and controls, and descending normalized latency (lower panel) of variants and controls showing (residual) luminescence responses in the bioassay. (B) Scatter plot showing the distribution of the different variants and controls. (A to B) Benign variants identified from gnomAD are depicted as dark blue triangles and the CNGA1/A3 homology control variants as light blue triangles. The 30 CNGA1 variants that were classified as LP by ACMG/AMP criteria prior to functional testing are depicted as red squared. The 56 CNGA1 variants categorized as VUS without the functional data are grouped depending on the functional outcome. Dotted lines indicate z-scores based thresholds of ±2.56xSD, corresponding to p≤0.01 and based on the benign variants and CNGA1/A3-homology control variants. For A to B, the mean ± SD of at least three independent transfections (biological replicates) is shown.


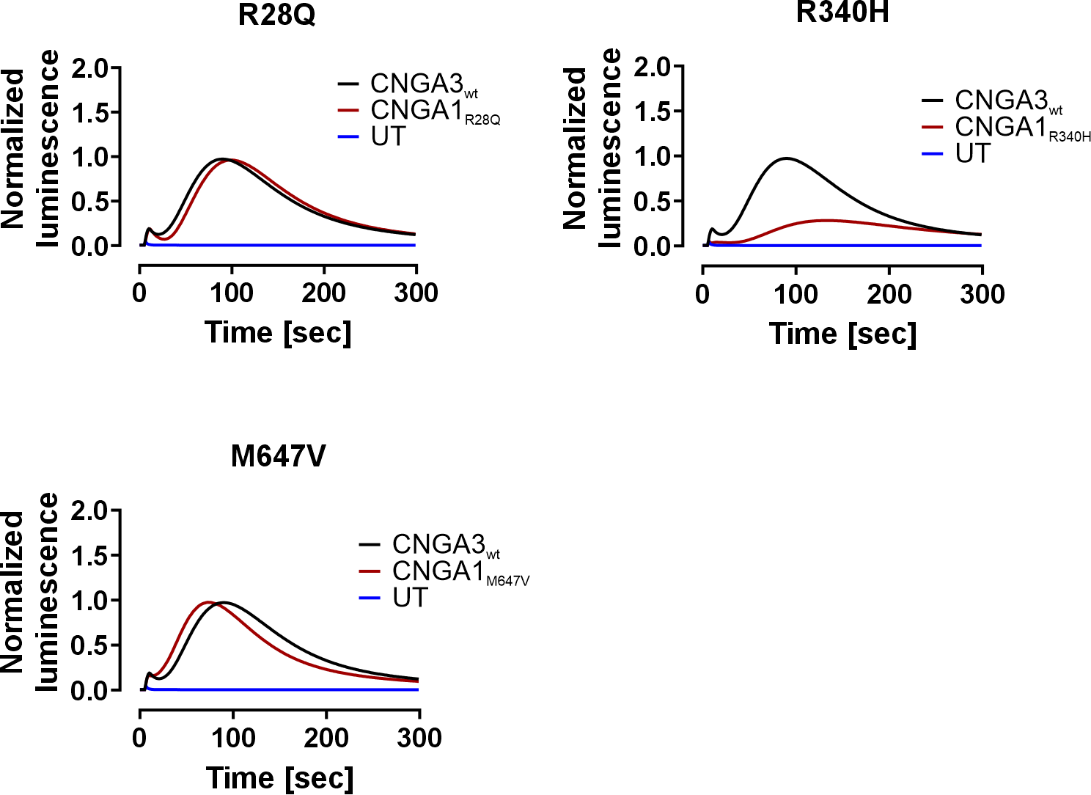


Supplementary figure 5: Normalized luminescence traces of benign CNGA1 variants identified from gnomAD. Untransfected HEK293^aequo^ cells (blue) and cells expressing CNGA3_wt_ channels (black) are shown as controls. Mean data of at least three biological replicates are illustrated. UT: untransfected; WT: wild-type.


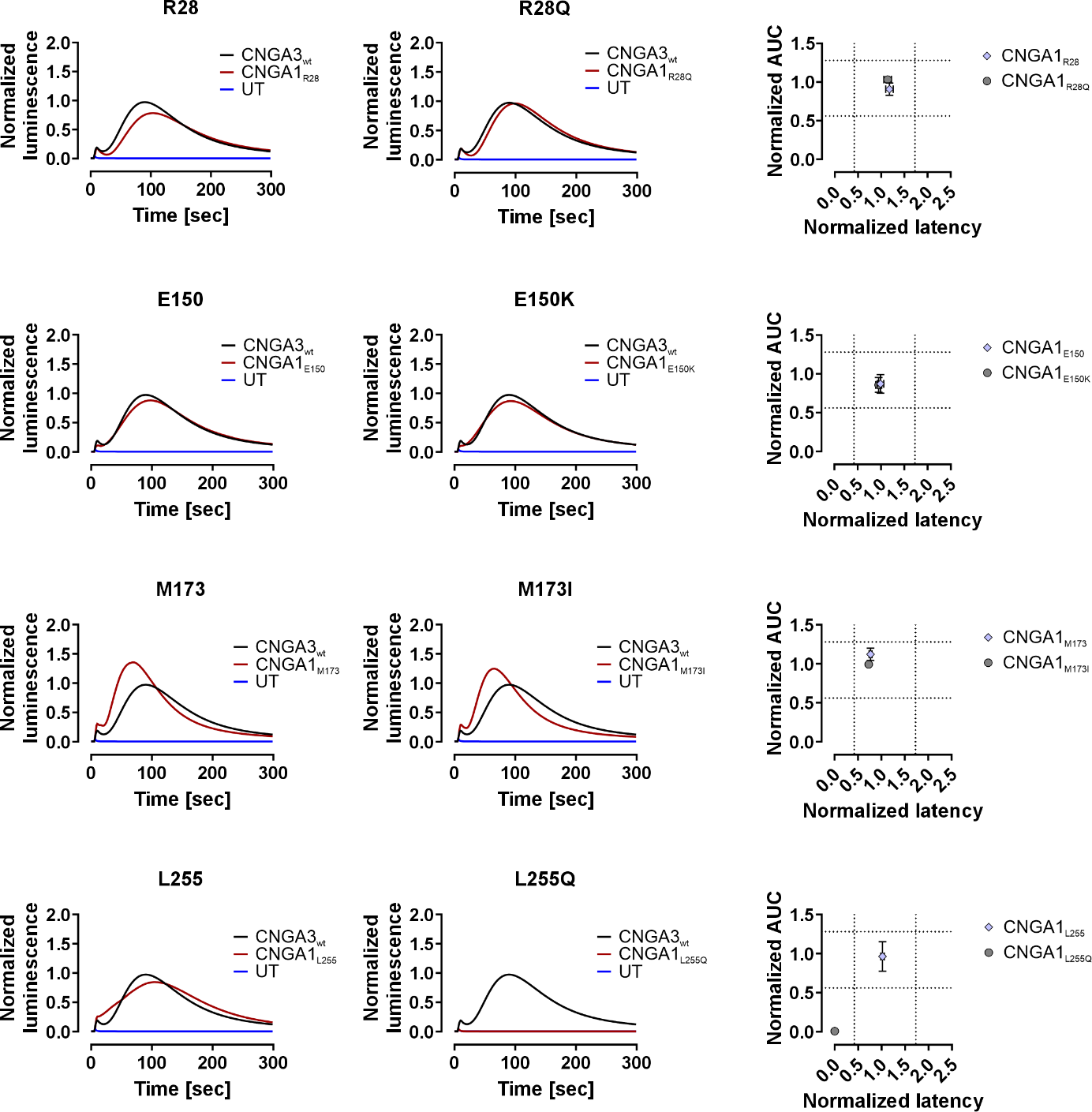


(Supplementary figure 6)


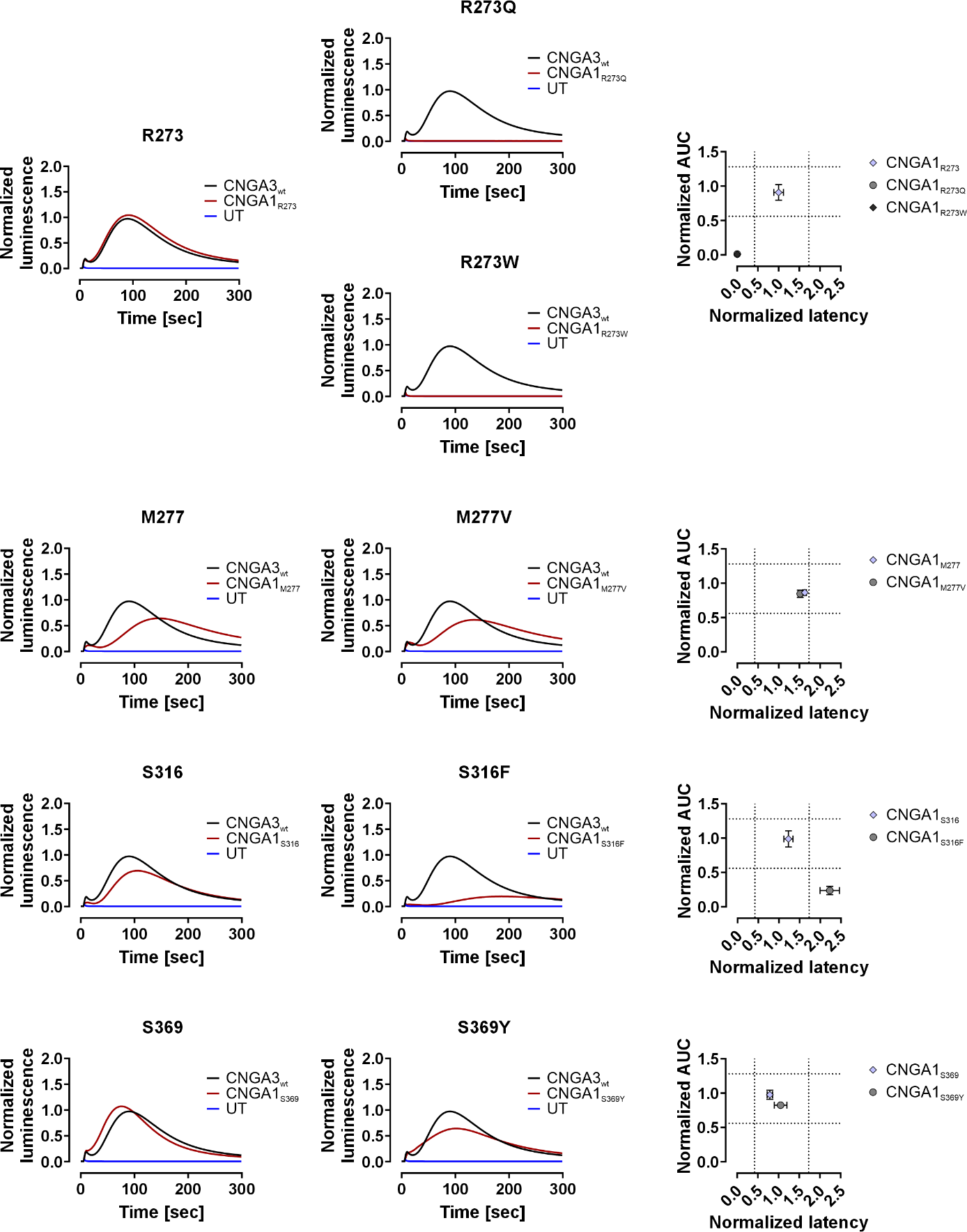


(Supplementary figure 6 continued)


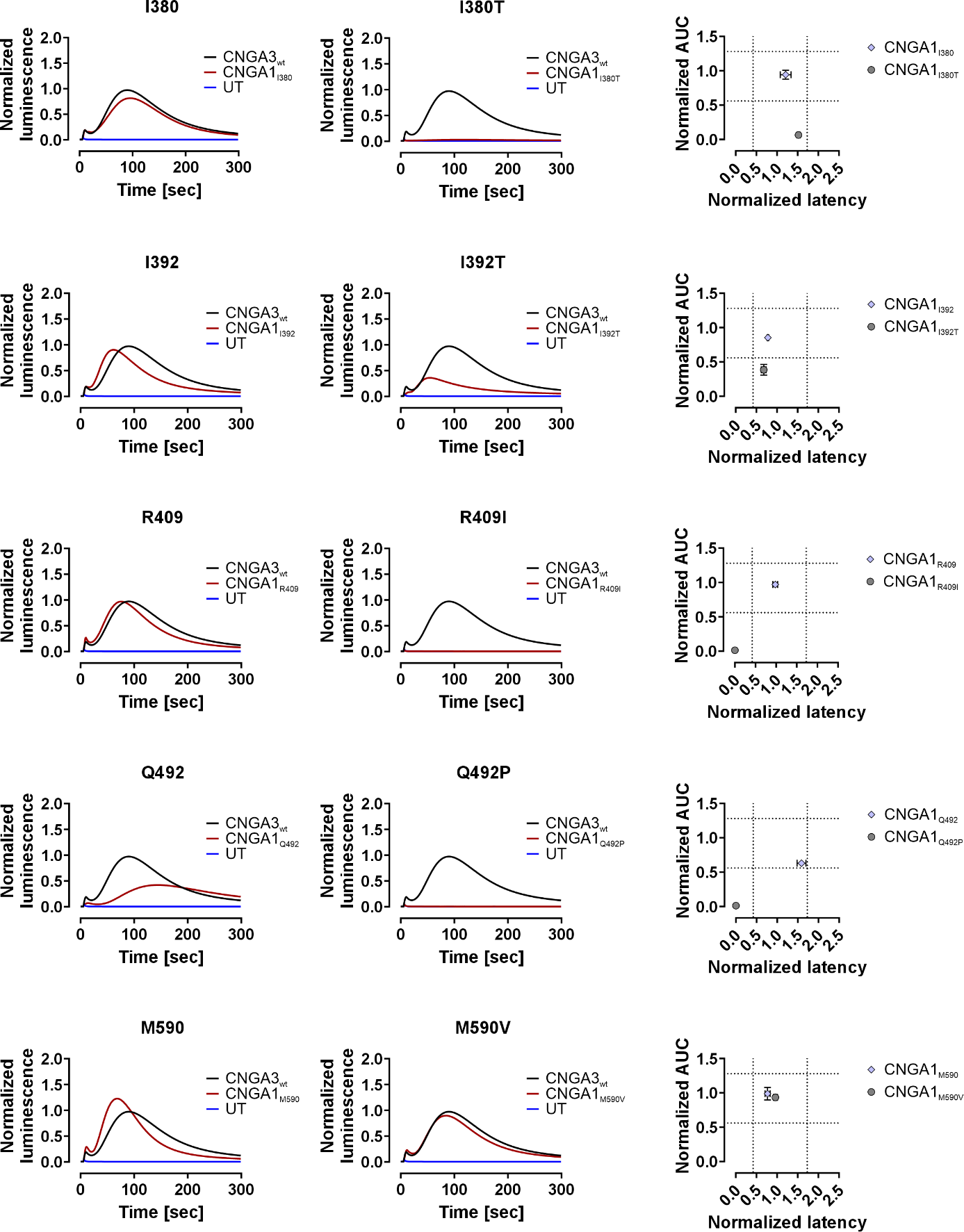


(Supplementary figure 6 continued)


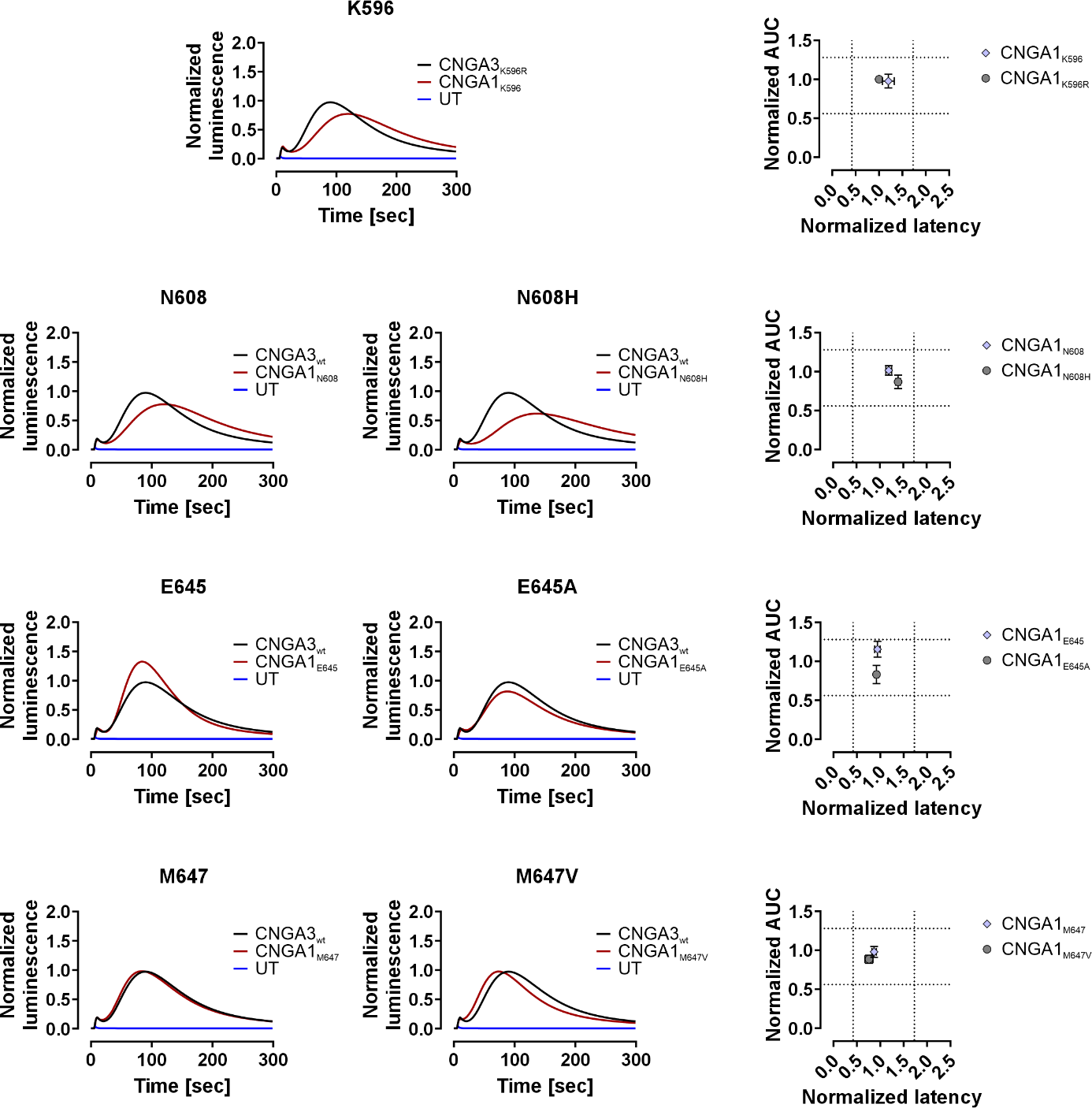


Supplementary figure 6: Luminescence responses of CNGA1 variant channels and their corresponding CNGA1/A3 homology control variant channels for amino acid positions not conserved between human CNGA1 and CNGA3. First column: normalized luminescence signals of CNGA3 wild-type and CNGA1/A3 homology control variants. Second column: normalized luminescence signals of CNGA3 wild-type and CNGA1 variant channel are displayed. The luminescence trace for the CNGA1 variant p.K596R corresponds to the CNGA3wt trace. The third column represents scatterplots of the normalized latency versus AUC for the variants and the control. Dotted lines indicated z-score based thresholds to differentiate functionally normal and abnormal responses. In the first two columns averaged luminescence traces are presented and mean ± SD is shown in the scatterplot. At least three independent transfections (biological replicates) were analyzed. UT: untransfected; WT: wild-type.


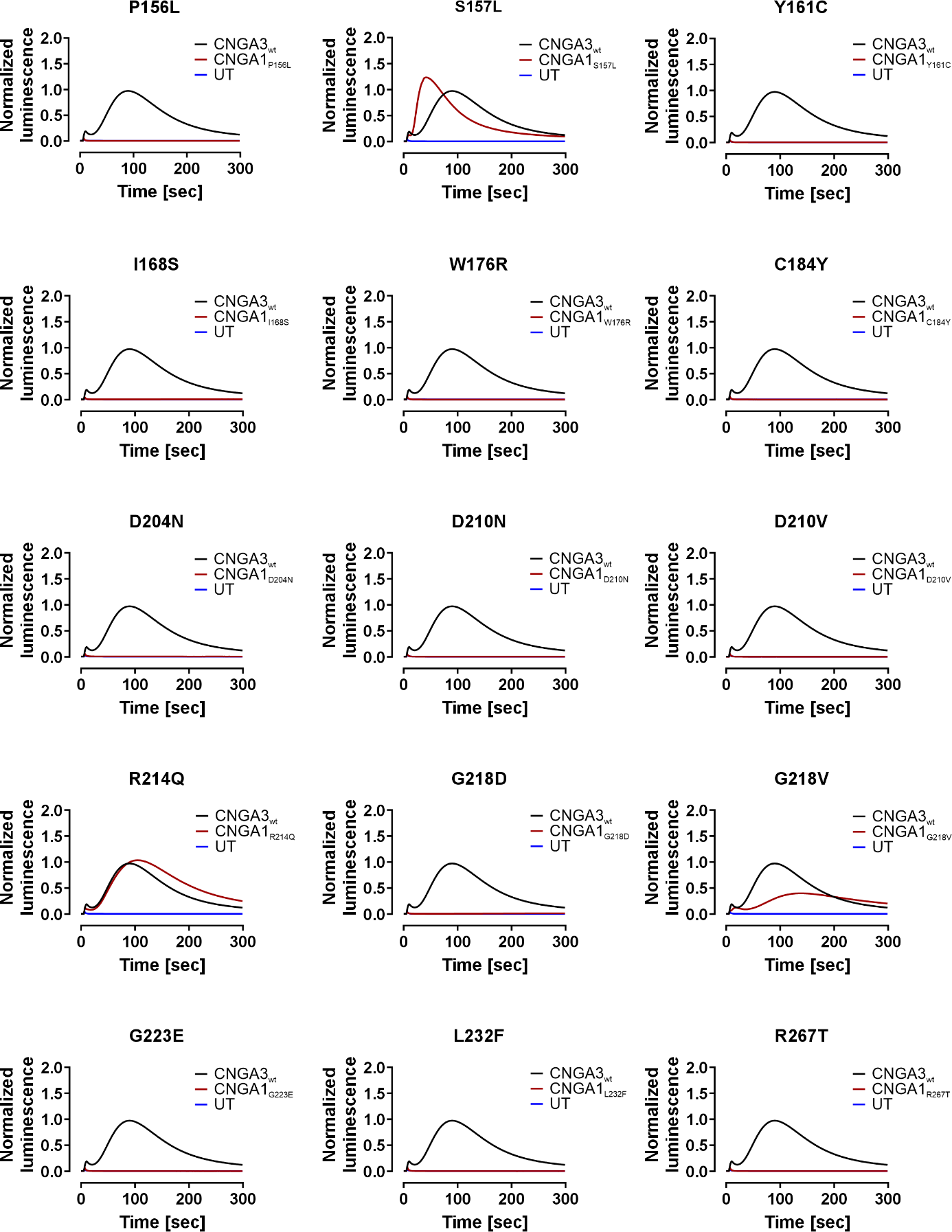


(Supplementary figure 7)


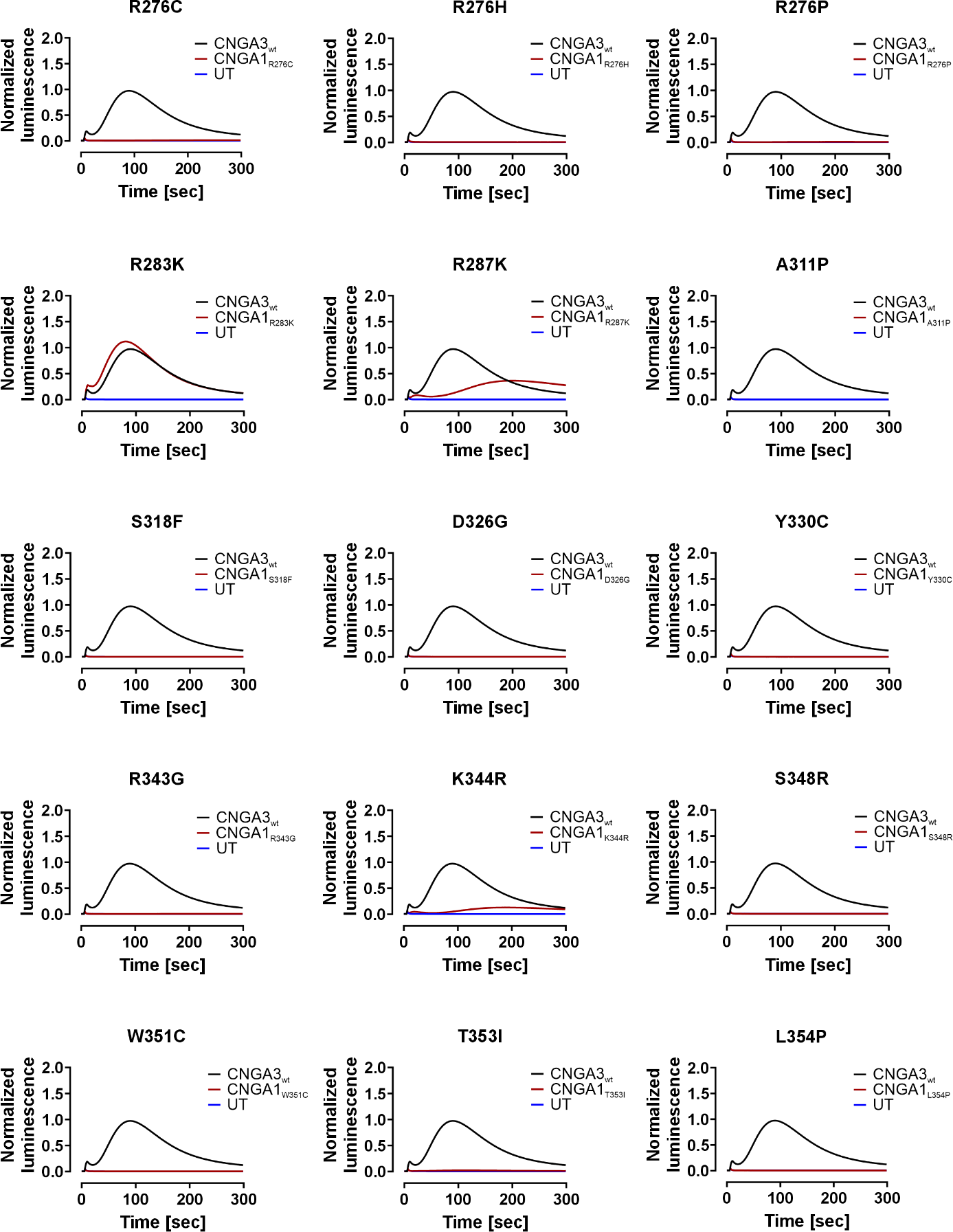


(Supplementary figure 7 continued)

**
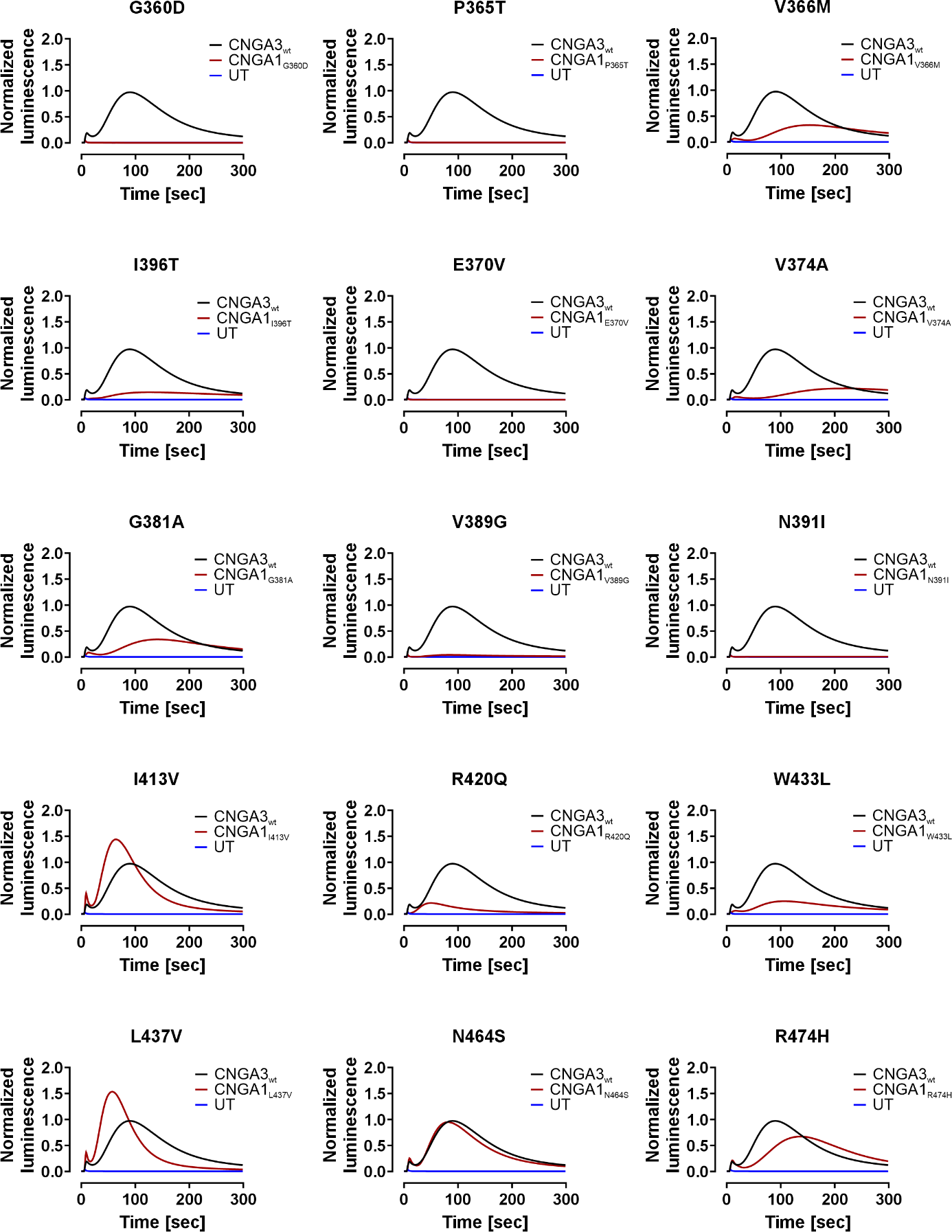
**

(Supplementary figure 7 continued)

**
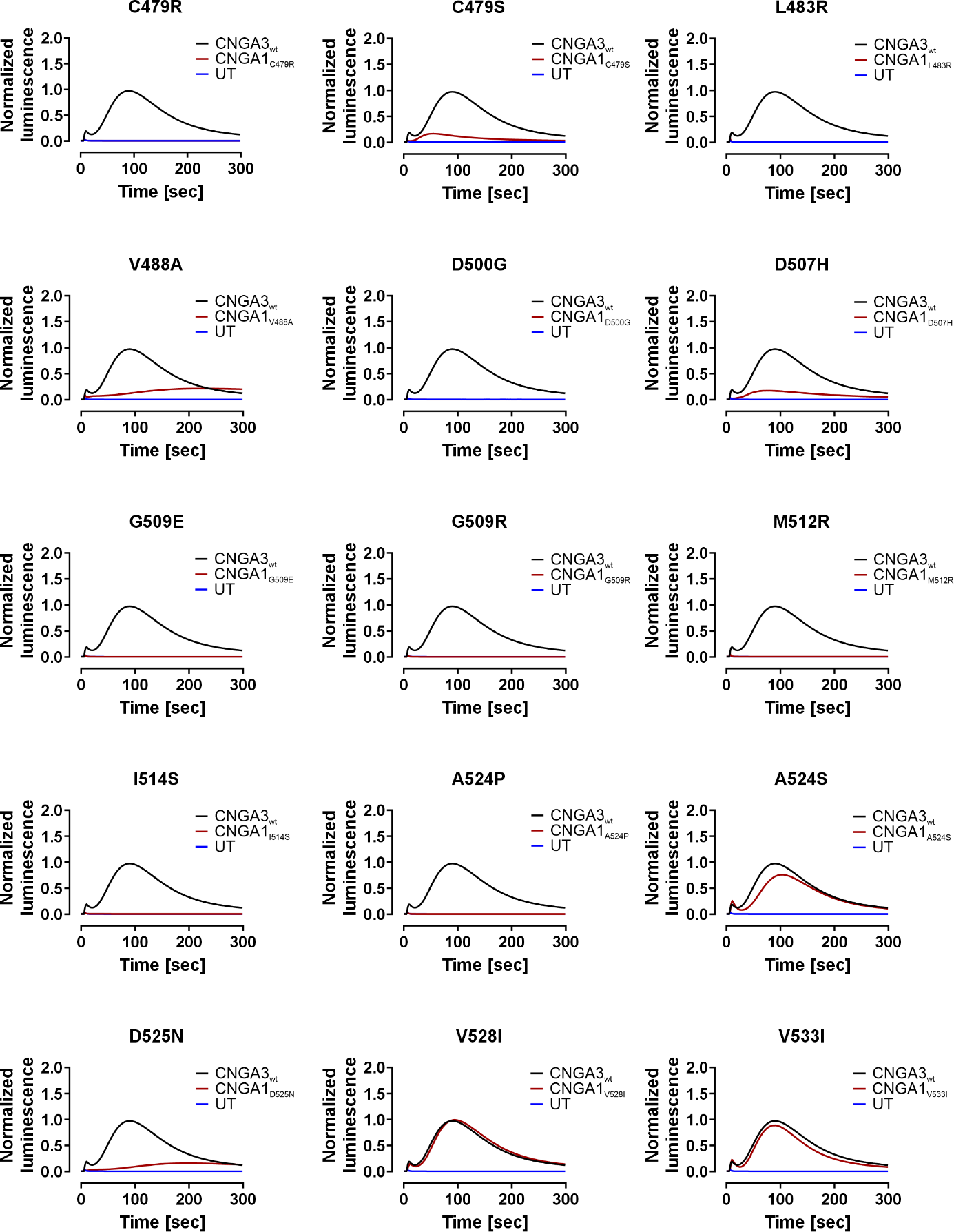
**

(Supplementary figure 7 continued)

**
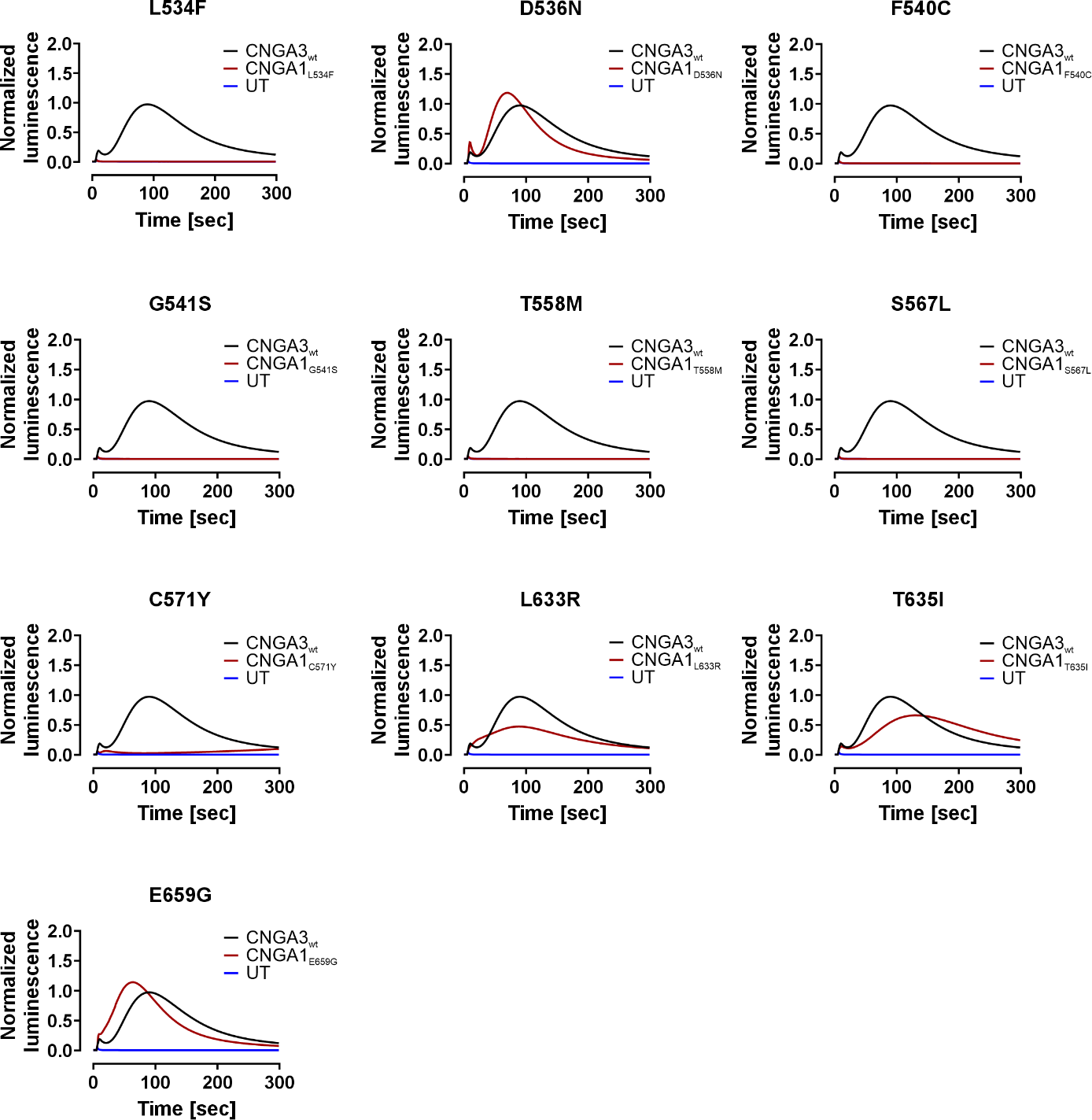
**

Supplementary figure 7: Normalized luminescence responses for CNGA1 variants affecting amino acid positions conserved between human CNGA1 and CNGA3. Wild-type CNGA3 channels and untransfected cells served as controls. At least three independent transfections (biological replicates) were analyzed. UT: untransfected; WT: wild-type


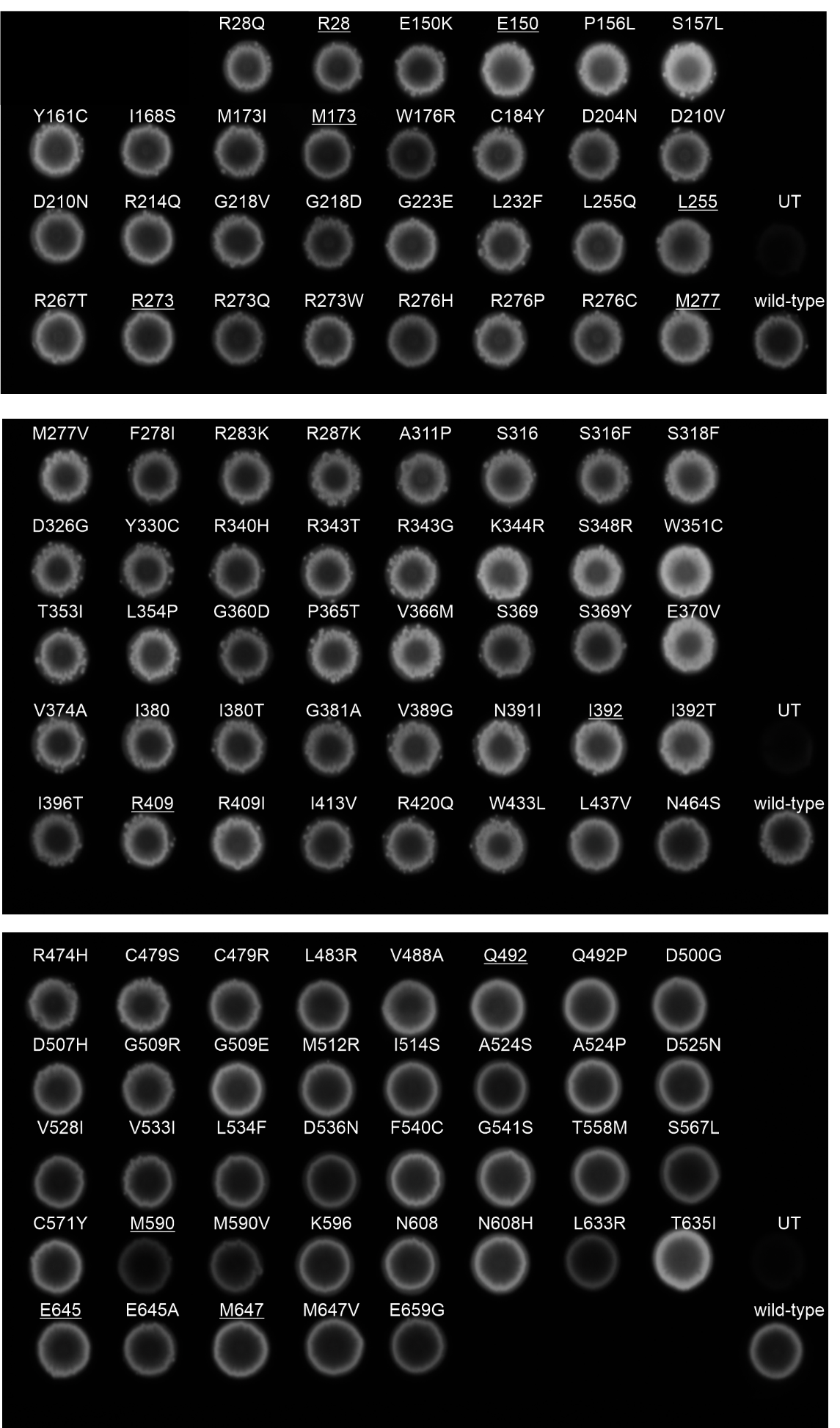


Supplementary figure 8: Dot blot analysis to confirm heterologous expression of the CNGA1 variant channels in HEK293^aequo^ cells. Untransfected cells and cells expressing wild-type CNGA3 served as controls. A total of 5 µg of whole-cell lysate – pooled from three independent transfections – was dotted per sample. CNGA1/A3-homology control variants are underlined. UT: untransfected.


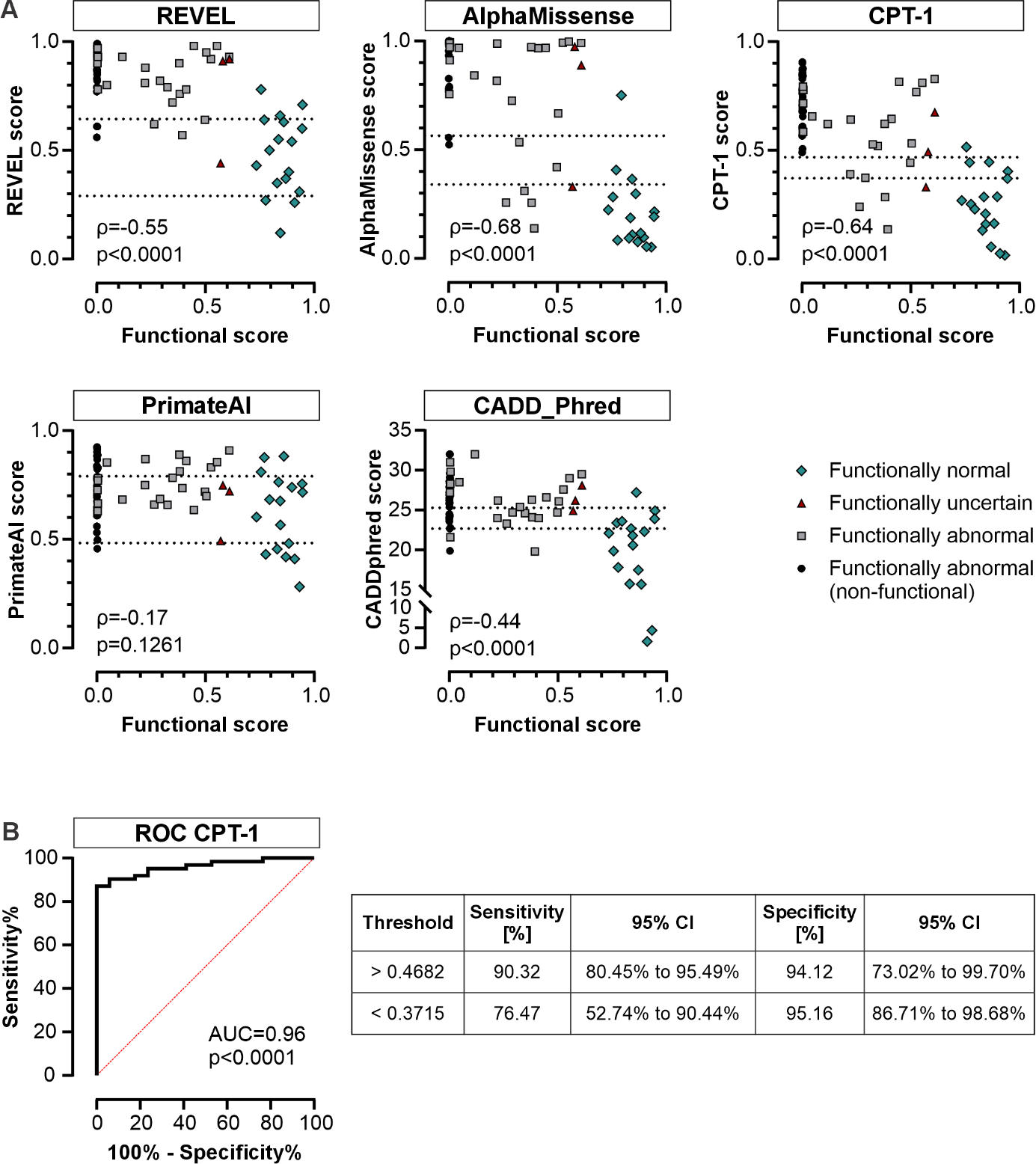


Supplementary figure 9: Spearman correlation of the functional data and variant prediction scores of five different variant effect predictor (VEP) tools. (A) For easier evaluation, the parameter AUC and latency, that were assessed with the luminescence-based bioassay, were combined into a functional score as described in the material and methods section. The Spearman correlation coefficients (ρ) of the functional scores and the VEP scores were calculated for all 82 CNGA1 missense variants outside the CLZ. Dotted lines indicate thresholds used for the computational tool to predict a variant effect as benign, uncertain or pathogenic following the recommendations from (Jagota et al., 2023; Pejaver et al., 2022), https://alphamissense.hegelab.org or were determined via Receiver operating characteristics (ROC; see B). (B) ROC analysis to establish thresholds for CPT-1 to differentiate benign from pathogenic variants. The area under the ROC (AUC) as well as the selected thresholds with approximately 95 % specificity are provided.


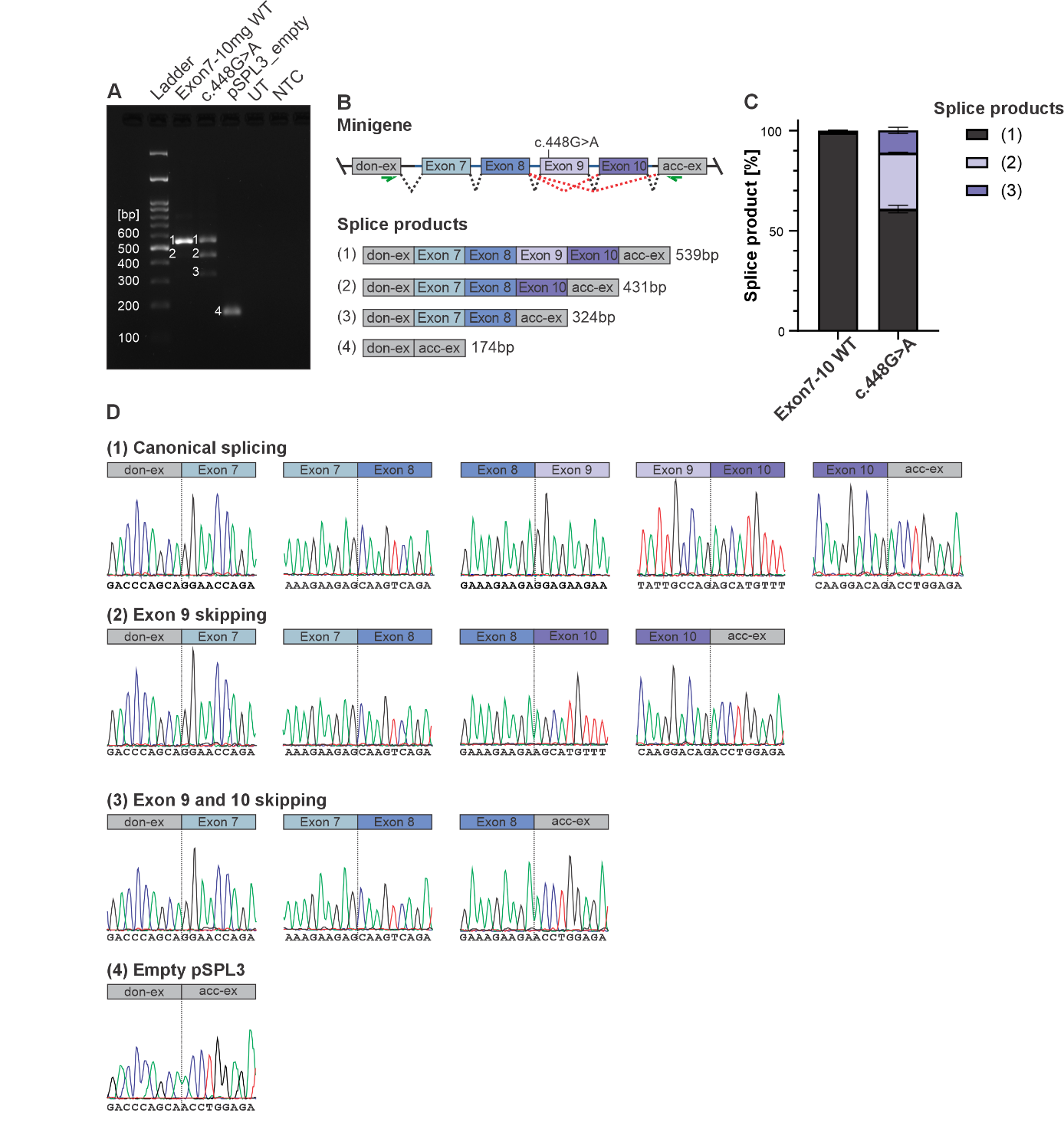


Supplementary figure 10: *In vitro* minigene splice assay to assess *CNGA1* variant c.448G>A. (A) Agarose gel image of RT-PCRs from HEK293T/17 cells transfected with the wild-type or mutant CNGA1_ex7-10 minigenes. Cells transfected with the empty pSPL3 and untransfected cells served as controls. Numbers indicate the observed splice products, whose composition is illustrated in (B). (B) Composition of the CNGA1_ex7-10mg and location of the studied variant is depicted in the upper panel. Dotted lines underneath the minigene represent correct (black) or missplicing events (red). In the lower panel, the composition of the observed spliced transcripts and the expected RT-PCR fragment size using don-ex and acc-ex specific primers (green arrows) is presented. (C) Relative quantification of splice products from agarose gels. Identity of the splice products is depicted in (B). Two independent transfections, RNA isolations, cDNA synthesis and RT-PCRs were studied. Data are presented as mean ± SD. (D) Electropherograms showing the splice junctions of the identified splice products. Acc-ex: pSPL3 acceptor exon; Don-ex: pSPL3 donor exon; NTC: no template control; UT: Untransfected; WT: wild-type.


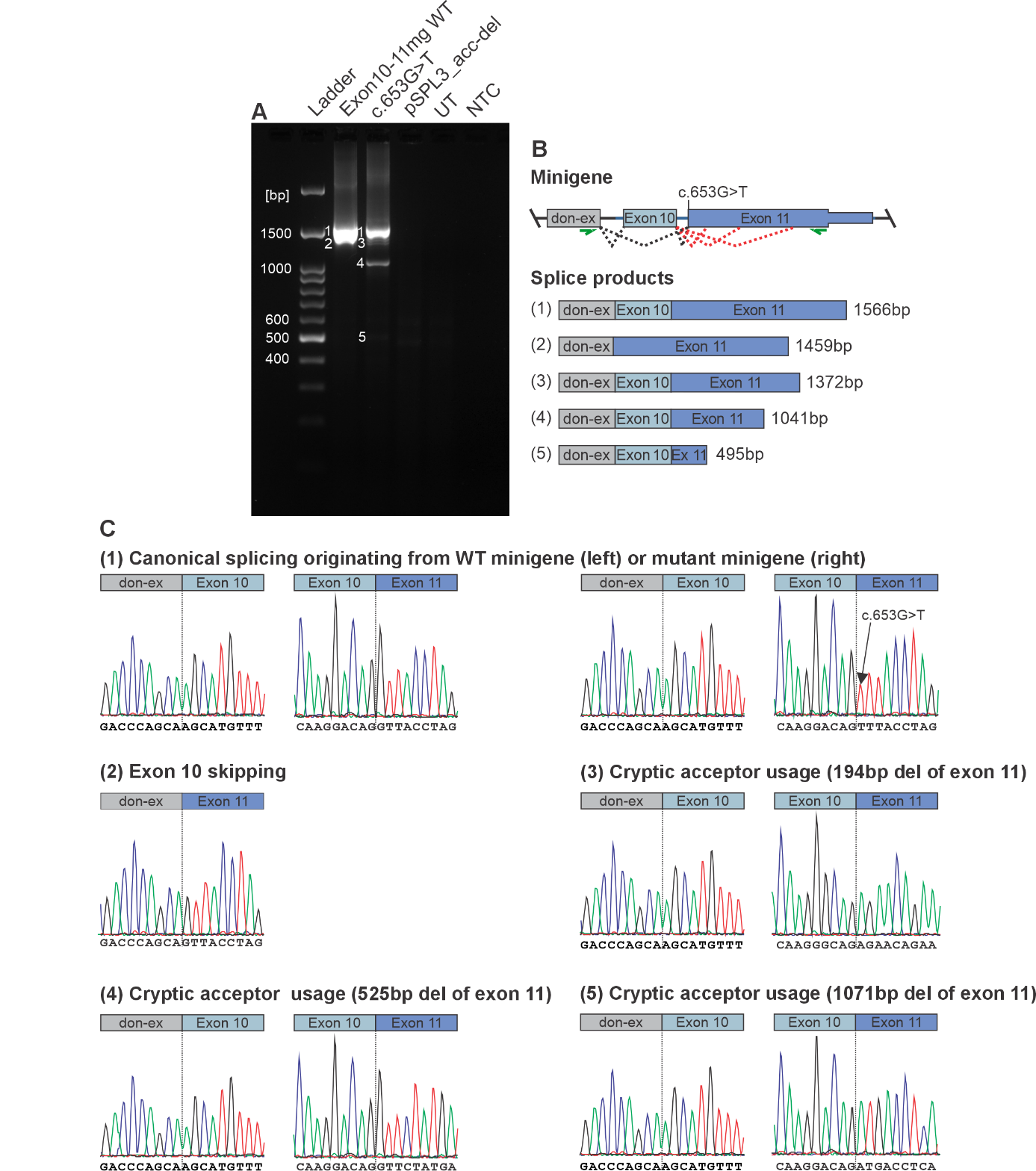


Supplementary figure 11: *In vitro* minigene splice assay to assess *CNGA1* variant c.653G>T. (A) Agarose gel image of RT-PCRs from HEK293T/17 cells transfected with the wild-type or mutant CNGA1_ex10-11 minigenes. Cells transfected with the empty pSPL3_acc-del and untransfected cells served as controls. Numbers indicate the observed splice products, whose composition is illustrated in (B). (B) Composition of the CNGA1_ex10-11mg and location of the studied variant is depicted in the upper panel. Dotted lines underneath the minigene represent correct (black) or missplicing events (red). Location of primers (green arrows) used for the RT-PCR are indicted below the minigene. In the lower panel, the composition of the observed spliced transcripts and the expected RT-PCR fragment size is presented. (C) Electropherograms showing the splice junctions of the identified splice products. Only splice product that were detected in at least two clones (2/26) after subcloning of RT-PCR products are depicted. Don-ex: pSPL3 donor exon; NTC: no template control; UT: untransfected; WT: wild-type.

**References**

Birtel, J., Gliem, M., Mangold, E., Muller, P. L., Holz, F. G., Neuhaus, C., Lenzner, S., Zahnleiter, D., Betz, C., Eisenberger, T., Bolz, H. J., & Charbel Issa, P. (2018). Next-generation sequencing identifies unexpected genotype-phenotype correlations in patients with retinitis pigmentosa. PLoS One, 13(12), e0207958. <https://doi.org/10.1371/journal.pone.0207958>

Brnich, S. E., Abou Tayoun, A. N., Couch, F. J., Cutting, G. R., Greenblatt, M. S., Heinen, C. D., Kanavy, D. M., Luo, X., McNulty, S. M., Starita, L. M., Tavtigian, S. V., Wright, M. W., Harrison, S. M., Biesecker, L. G., Berg, J. S., & Clinical Genome Resource Sequence Variant Interpretation Working, G. (2019). Recommendations for application of the functional evidence PS3/BS3 criterion using the ACMG/AMP sequence variant interpretation framework. Genome Med, 12(1), 3. https://doi.org/10.1186/s13073-019-0690-2

Carss, K. J., Arno, G., Erwood, M., Stephens, J., Sanchis-Juan, A., Hull, S., Megy, K., Grozeva, D., Dewhurst, E., Malka, S., Plagnol, V., Penkett, C., Stirrups, K., Rizzo, R., Wright, G., Josifova, D., Bitner-Glindzicz, M., Scott, R. H., Clement, E.,…Raymond, F. L. (2017). Comprehensive Rare Variant Analysis via Whole-Genome Sequencing to Determine the Molecular Pathology of Inherited Retinal Disease. *Am J Hum Genet*, *100*(1), 75-90. https://doi.org/10.1016/j.ajhg.2016.12.003

Comander, J., Weigel-DiFranco, C., Maher, M., Place, E., Wan, A., Harper, S., Sandberg, M. A., Navarro-Gomez, D., & Pierce, E. A. (2017). The Genetic Basis of Pericentral Retinitis Pigmentosa-A Form of Mild Retinitis Pigmentosa. *Genes (Basel)*, *8*(10). https://doi.org/10.3390/genes8100256

Costa, K. A., Salles, M. V., Whitebirch, C., Chiang, J., & Sallum, J. M. F. (2017). Gene panel sequencing in Brazilian patients with retinitis pigmentosa. *Int J Retina Vitreous*, *3*, 33. https://doi.org/10.1186/s40942-017-0087-6

Dan, H., Huang, X., Xing, Y., & Shen, Y. (2020). Application of targeted panel sequencing and whole exome sequencing for 76 Chinese families with retinitis pigmentosa. *Mol Genet Genomic Med*, *8*(3), e1131. https://doi.org/10.1002/mgg3.1131

Demir, S., Ates, E. A., Sevik, O., Sozer, B., Kose, T., Sahin, O., Arman, A., & Geckinli, B. B. (2025). Decoding the Genetic Puzzle of Inherited Retinal Dystrophies: Novel Insights From a Turkish Cohort. *Clin Genet*. <https://doi.org/10.1111/cge.14769>

Dryja, T. P., Finn, J. T., Peng, Y. W., McGee, T. L., Berson, E. L., & Yau, K. W. (1995). Mutations in the gene encoding the alpha subunit of the rod cGMP-gated channel in autosomal recessive retinitis pigmentosa. Proc Natl Acad Sci U S A, 92(22), 10177–10181. https://doi.org/10.1073/pnas.92.22.10177

Eisenberger, T., Neuhaus, C., Khan, A. O., Decker, C., Preising, M. N., Friedburg, C., Bieg, A., Gliem, M., Charbel Issa, P., Holz, F. G., Baig, S. M., Hellenbroich, Y., Galvez, A., Platzer, K., Wollnik, B., Laddach, N., Ghaffari, S. R., Rafati, M., Botzenhart, E.,…Bolz, H. J. (2013). Increasing the yield in targeted next-generation sequencing by implicating CNV analysis, non-coding exons and the overall variant load: the example of retinal dystrophies. *PLoS One*, *8*(11), e78496. https://doi.org/10.1371/journal.pone.0078496

Ellingford, J. M., Barton, S., Bhaskar, S., O'Sullivan, J., Williams, S. G., Lamb, J. A., Panda, B., Sergouniotis, P. I., Gillespie, R. L., Daiger, S. P., Hall, G., Gale, T., Lloyd, I. C., Bishop, P. N., Ramsden, S. C., & Black, G. C. M. (2016). Molecular findings from 537 individuals with inherited retinal disease. *J Med Genet*, *53*(11), 761-767. https://doi.org/10.1136/jmedgenet-2016-103837

Gao, F. J., Li, J. K., Chen, H., Hu, F. Y., Zhang, S. H., Qi, Y. H., Xu, P., Wang, D. D., Wang, L. S., Chang, Q., Zhang, Y. J., Liu, W., Li, W., Wang, M., Chen, F., Xu, G. Z., & Wu, J. H. (2019). Genetic and Clinical Findings in a Large Cohort of Chinese Patients with Suspected Retinitis Pigmentosa. *Ophthalmology*, *126*(11), 1549-1556. https://doi.org/10.1016/j.ophtha.2019.04.038

Gao, Q., Liu, Y., Lei, X., Deng, Q., Tong, Y., Du, L., & Shen, Y. (2019). A Novel CNGA1 Gene Mutation (c.G622A) of Autosomal Recessive Retinitis Pigmentosa Leads to the CNGA1 Protein Reduction on Membrane. *Biochem Genet*, *57*(4), 540-554. https://doi.org/10.1007/s10528-019-09907-3

Gonzalez-del Pozo, M., Borrego, S., Barragan, I., Pieras, J. I., Santoyo, J., Matamala, N., Naranjo, B., Dopazo, J., & Antinolo, G. (2011). Mutation screening of multiple genes in Spanish patients with autosomal recessive retinitis pigmentosa by targeted resequencing. *PLoS One*, *6*(12), e27894. https://doi.org/10.1371/journal.pone.0027894

Goto, K., Koyanagi, Y., Akiyama, M., Murakami, Y., Fukushima, M., Fujiwara, K., Iijima, H., Yamaguchi, M., Endo, M., Hashimoto, K., Ishizu, M., Hirakata, T., Mizobuchi, K., Takayama, M., Ota, J., Sajiki, A. F., Kominami, T., Ushida, H., Fujita, K.,…Nishiguchi, K. M. (2024). Disease-specific variant interpretation highlighted the genetic findings in 2325 Japanese patients with retinitis pigmentosa and allied diseases. *J Med Genet*, *61*(7), 613-620. https://doi.org/10.1136/jmg-2023-109750

Hayman, T., Millo, T., Hendler, K., Chowers, I., Gross, M., Banin, E., & Sharon, D. (2024). Whole exome sequencing of 491 individuals with inherited retinal diseases reveals a large spectrum of variants and identification of novel candidate genes. *J Med Genet*, *61*(3), 224-231. https://doi.org/10.1136/jmg-2023-109482

Heutinck, P. A. T., van den Born, L. I., Vermeer, M., Iglesias Gonzales, A. I., Hoyng, C. B., Pott, J. W. R., Kroes, H. Y., van Schooneveld, M. J., Boon, C. J. F., van Genderen, M. M., Plomp, A. S., de Jong-Hesse, Y., van Egmond-Ebbeling, M. B., Hoefsloot, L. H., A, A. B., Klaver, C. C. W., Meester-Smoor, M. A., Thiadens, A., & Verhoeven, V. J. M. (2024). Frequency and Genetic Spectrum of Inherited Retinal Dystrophies in a Large Dutch Pediatric Cohort: The RD5000 Consortium. *Invest Ophthalmol Vis Sci*, *65*(10), 40. https://doi.org/10.1167/iovs.65.10.40

Huang, X. F., Huang, F., Wu, K. C., Wu, J., Chen, J., Pang, C. P., Lu, F., Qu, J., & Jin, Z. B. (2015). Genotype-phenotype correlation and mutation spectrum in a large cohort of patients with inherited retinal dystrophy revealed by next-generation sequencing. Genet Med, 17(4), 271–278. https://doi.org/10.1038/gim.2014.138

Jagota, M., Ye, C., Albors, C., Rastogi, R., Koehl, A., Ioannidis, N., & Song, Y. S. (2023). Cross-protein transfer learning substantially improves disease variant prediction. *Genome Biol*, *24*(1), 182. https://doi.org/10.1186/s13059-023-03024-6

Jiang, L., Liang, X., Li, Y., Wang, J., Zaneveld, J. E., Wang, H., Xu, S., Wang, K., Wang, B., Chen, R., & Sui, R. (2015). Comprehensive molecular diagnosis of 67 Chinese Usher syndrome probands: high rate of ethnicity specific mutations in Chinese USH patients. Orphanet J Rare Dis, 10, 110. <https://doi.org/10.1186/s13023-015-0329-3>

Jin, Z. B., Mandai, M., Yokota, T., Higuchi, K., Ohmori, K., Ohtsuki, F., Takakura, S., Itabashi, T., Wada, Y., Akimoto, M., Ooto, S., Suzuki, T., Hirami, Y., Ikeda, H., Kawagoe, N., Oishi, A., Ichiyama, S., Takahashi, M., Yoshimura, N., & Kosugi, S. (2008). Identifying pathogenic genetic background of simplex or multiplex retinitis pigmentosa patients: a large scale mutation screening study. J Med Genet, 45(7), 465–472. <https://doi.org/10.1136/jmg.2007.056416>

Jin, X., Qu, L. H., Hou, B. K., Xu, H. W., Meng, X. H., Pang, C. P., & Yin, Z. Q. (2016). Novel compound heterozygous mutation in the CNGA1 gene underlie autosomal recessive retinitis pigmentosa in a Chinese family. Biosci Rep, 36(1), e00289. https://doi.org/10.1042/BSR20150131

Jespersgaard, C., Fang, M., Bertelsen, M., Dang, X., Jensen, H., Chen, Y., Bech, N., Dai, L., Rosenberg, T., Zhang, J., Moller, L. B., Tumer, Z., Brondum-Nielsen, K., & Gronskov, K. (2019). Molecular genetic analysis using targeted NGS analysis of 677 individuals with retinal dystrophy. *Sci Rep*, *9*(1), 1219. <https://doi.org/10.1038/s41598-018-38007-2>

Kandaswamy, S., Zobel, L., John, B., Santhiya, S. T., Bogedein, J., Przemeck, G. K. H., Gailus-Durner, V., Fuchs, H., Biel, M., de Angelis, M. H., Graw, J., Michalakis, S., & Amarie, O. V. (2022). Mutations within the cGMP-binding domain of CNGA1 causing autosomal recessive retinitis pigmentosa in human and animal model. Cell Death Discov, 8(1), 387. https://doi.org/10.1038/s41420-022-01185-0

Karali, M., Testa, F., Di Iorio, V., Torella, A., Zeuli, R., Scarpato, M., Romano, F., Onore, M. E., Pizzo, M., Melillo, P., Brunetti-Pierri, R., Passerini, I., Pelo, E., Cremers, F. P. M., Esposito, G., Nigro, V., Simonelli, F., & Banfi, S. (2022). Genetic epidemiology of inherited retinal diseases in a large patient cohort followed at a single center in Italy. *Sci Rep*, *12*(1), 20815. https://doi.org/10.1038/s41598-022-24636-1

Kars, M. E., Basak, A. N., Onat, O. E., Bilguvar, K., Choi, J., Itan, Y., Caglar, C., Palvadeau, R., Casanova, J. L., Cooper, D. N., Stenson, P. D., Yavuz, A., Bulus, H., Gunel, M., Friedman, J. M., & Ozcelik, T. (2021). The genetic structure of the Turkish population reveals high levels of variation and admixture. *Proc Natl Acad Sci U S A*, *118*(36). https://doi.org/10.1073/pnas.2026076118

Katagiri, S., Akahori, M., Sergeev, Y., Yoshitake, K., Ikeo, K., Furuno, M., Hayashi, T., Kondo, M., Ueno, S., Tsunoda, K., Shinoda, K., Kuniyoshi, K., Tsurusaki, Y., Matsumoto, N., Tsuneoka, H., & Iwata, T. (2014). Whole exome analysis identifies frequent CNGA1 mutations in Japanese population with autosomal recessive retinitis pigmentosa. *PLoS One*, *9*(9), e108721. https://doi.org/10.1371/journal.pone.0108721

Koyanagi, Y., Akiyama, M., Nishiguchi, K. M., Momozawa, Y., Kamatani, Y., Takata, S., Inai, C., Iwasaki, Y., Kumano, M., Murakami, Y., Omodaka, K., Abe, T., Komori, S., Gao, D., Hirakata, T., Kurata, K., Hosono, K., Ueno, S., Hotta, Y.,…Sonoda, K. H. (2019). Genetic characteristics of retinitis pigmentosa in 1204 Japanese patients. *J Med Genet*, *56*(10), 662-670. https://doi.org/10.1136/jmedgenet-2018-105691

Lieviant, J. A., Chan, C. M., Bylstra, Y., Jain, K., Teo, J. X., Lim, W. W., Kam, S., Chao, T. W., Chai Bin Siew, N., Davila, S., Vithana, E. N., Mathur, R. S., Tan, T. E., Tan, P., Jamuar, S. S., Fenner, B. J., & Lim, W. K. (2025). Determinants of diagnostic yield in a multi-ethnic Asian inherited retinal disease cohort. *Eur J Hum Genet*. https://doi.org/10.1038/s41431-025-01833-w

Lin, S., Vermeirsch, S., Pontikos, N., Martin-Gutierrez, M. P., Daich Varela, M., Malka, S., Schiff, E., Knight, H., Wright, G., Jurkute, N., Simcoe, M. J., Yu-Wai-Man, P., Moosajee, M., Michaelides, M., Mahroo, O. A., Webster, A. R., & Arno, G. (2024). Spectrum of Genetic Variants in the Most Common Genes Causing Inherited Retinal Disease in a Large Molecularly Characterized United Kingdom Cohort. *Ophthalmol Retina*, *8*(7), 699-709. https://doi.org/10.1016/j.oret.2024.01.012

Liu, X., Tao, T., Zhao, L., Li, G., & Yang, L. (2021). Molecular diagnosis based on comprehensive genetic testing in 800 Chinese families with non-syndromic inherited retinal dystrophies. *Clin Exp Ophthalmol*, *49*(1), 46-59. <https://doi.org/10.1111/ceo.13875>

Maeda, A., Yoshida, A., Kawai, K., Arai, Y., Akiba, R., Inaba, A., Takagi, S., Fujiki, R., Hirami, Y., Kurimoto, Y., Ohara, O., & Takahashi, M. (2018). Development of a molecular diagnostic test for Retinitis Pigmentosa in the Japanese population. Jpn J Ophthalmol, 62(4), 451–457. https://doi.org/10.1007/s10384-018-0601-x

Maria, M., Ajmal, M., Azam, M., Waheed, N. K., Siddiqui, S. N., Mustafa, B., Ayub, H., Ali, L., Ahmad, S., Micheal, S., Hussain, A., Shah, S. T., Ali, S. H., Ahmed, W., Khan, Y. M., den Hollander, A. I., Haer-Wigman, L., Collin, R. W., Khan, M. I.,…Cremers, F. P. (2015). Homozygosity mapping and targeted sanger sequencing reveal genetic defects underlying inherited retinal disease in families from pakistan. *PLoS One*, *10*(3), e0119806. https://doi.org/10.1371/journal.pone.0119806

Martinez-Francois, J. R., Xu, Y., & Lu, Z. (2009). Mutations reveal voltage gating of CNGA1 channels in saturating cGMP. *J Gen Physiol*, *134*(2), 151-164. https://doi.org/10.1085/jgp.200910240

Matczynska, E., Bec-Gajowniczek, M., Sivitskaya, L., Gregorczyk, E., Lyszkiewicz, P., Szymanczak, R., Jedrzejowska, M., Wylegala, E., Krawczynski, M. R., Teper, S., & Boguszewska-Chachulska, A. (2024). Optimised, Broad NGS Panel for Inherited Eye Diseases to Diagnose 1000 Patients in Poland. *Biomedicines*, *12*(6). https://doi.org/10.3390/biomedicines12061355

Mihalich, A., Cammarata, G., Tremolada, G., Manfredini, E., Bianchi Marzoli, S., & Di Blasio, A. M. (2024). Genetic Characterization of 191 Probands with Inherited Retinal Dystrophy by Targeted NGS Analysis. *Genes (Basel)*, *15*(6). https://doi.org/10.3390/genes15060766

Molday, R. S., Molday, L. L., Dose, A., Clark-Lewis, I., Illing, M., Cook, N. J., Eismann, E., & Kaupp, U. B. (1991). The cGMP-gated channel of the rod photoreceptor cell characterization and orientation of the amino terminus. *J Biol Chem*, *266*(32), 21917-21922. https://www.ncbi.nlm.nih.gov/pubmed/1718987

Muhammad, A., Calandranis, M. E., Li, B., Yang, T., Blackwell, D. J., Harvey, M. L., Smith, J. E., Daniel, Z. A., Chew, A. E., Capra, J. A., Matreyek, K. A., Fowler, D. M., Roden, D. M., & Glazer, A. M. (2024). High-throughput functional mapping of variants in an arrhythmia gene, KCNE1, reveals novel biology. Genome Med, 16(1), 73. https://doi.org/10.1186/s13073-024-01340-5

Oishi, M., Oishi, A., Gotoh, N., Ogino, K., Higasa, K., Iida, K., Makiyama, Y., Morooka, S., Matsuda, F., & Yoshimura, N. (2014). Comprehensive molecular diagnosis of a large cohort of Japanese retinitis pigmentosa and Usher syndrome patients by next-generation sequencing. *Invest Ophthalmol Vis Sci*, *55*(11), 7369-7375. https://doi.org/10.1167/iovs.14-15458

Ozguc Caliskan, B., Uslu, K., Sinim Kahraman, N., Erkilic, K., Oner, A., & Dundar, M. (2024). Beyond the phenotype: Exploring inherited retinal diseases with targeted next-generation sequencing in a Turkish cohort. *Clin Genet*, *106*(3), 258-266. <https://doi.org/10.1111/cge.14529>

Panneman, D. M., Hitti-Malin, R. J., Holtes, L. K., de Bruijn, S. E., Reurink, J., Boonen, E. G. M., Khan, M. I., Ali, M., Andreasson, S., De Baere, E., Banfi, S., Bauwens, M., Ben-Yosef, T., Bocquet, B., De Bruyne, M., de la Cerda, B., Coppieters, F., Farinelli, P., Guignard, T.,…Roosing, S. (2023). Cost-effective sequence analysis of 113 genes in 1,192 probands with retinitis pigmentosa and Leber congenital amaurosis. Front Cell Dev Biol, 11, 1112270.

Pejaver, V., Byrne, A. B., Feng, B. J., Pagel, K. A., Mooney, S. D., Karchin, R., O'Donnell-Luria, A., Harrison, S. M., Tavtigian, S. V., Greenblatt, M. S., Biesecker, L. G., Radivojac, P., Brenner, S. E., & ClinGen Sequence Variant Interpretation Working, G. (2022). Calibration of computational tools for missense variant pathogenicity classification and ClinGen recommendations for PP3/BP4 criteria. *Am J Hum Genet*, *109*(12), 2163-2177. https://doi.org/10.1016/j.ajhg.2022.10.013

Perea-Romero, I., Gordo, G., Iancu, I. F., Del Pozo-Valero, M., Almoguera, B., Blanco-Kelly, F., Carreno, E., Jimenez-Rolando, B., Lopez-Rodriguez, R., Lorda-Sanchez, I., Martin-Merida, I., Perez de Ayala, L., Riveiro-Alvarez, R., Rodriguez-Pinilla, E., Tahsin-Swafiri, S., Trujillo-Tiebas, M. J., Group, E. S., Group, E. S., Associated Clinical Study, G.,…Ayuso, C. (2021). Genetic landscape of 6089 inherited retinal dystrophies affected cases in Spain and their therapeutic and extended epidemiological implications. *Sci Rep*, *11*(1), 1526. <https://doi.org/10.1038/s41598-021-81093-y>

Perez-Carro, R., Corton, M., Sanchez-Navarro, I., Zurita, O., Sanchez-Bolivar, N., Sanchez-Alcudia, R., Lelieveld, S. H., Aller, E., Lopez-Martinez, M. A., Lopez-Molina, M. I., Fernandez-San Jose, P., Blanco-Kelly, F., Riveiro-Alvarez, R., Gilissen, C., Millan, J. M., Avila-Fernandez, A., & Ayuso, C. (2016). Panel-based NGS Reveals Novel Pathogenic Mutations in Autosomal Recessive Retinitis Pigmentosa. Sci Rep, 6, 19531. https://doi.org/10.1038/srep19531

Quinodoz, M., Kaminska, K., Cancellieri, F., Han, J. H., Peter, V. G., Celik, E., Janeschitz-Kriegl, L., Scharer, N., Hauenstein, D., Gyorgy, B., Calzetti, G., Hahaut, V., Custodio, S., Sousa, A. C., Wada, Y., Murakami, Y., Fernandez, A. A., Hernandez, C. R., Minguez, P.,…Rivolta, C. (2024). Detection of elusive DNA copy-number variations in hereditary disease and cancer through the use of noncoding and off-target sequencing reads. *Am J Hum Genet*, *111*(4), 701-713. https://doi.org/10.1016/j.ajhg.2024.03.001

Rehman, A. U., Peter, V. G., Quinodoz, M., Rashid, A., Khan, S. A., Superti-Furga, A., & Rivolta, C. (2019). Exploring the Genetic Landscape of Retinal Diseases in North-Western Pakistan Reveals a High Degree of Autozygosity and a Prevalent Founder Mutation in ABCA4. *Genes (Basel)*, *11*(1). https://doi.org/10.3390/genes11010012

Rho, S., Lee, H. M., Lee, K., & Park, C. (2000). Effects of mutation at a conserved N-glycosylation site in the bovine retinal cyclic nucleotide-gated ion channel. *FEBS Lett*, *478*(3), 246-252. https://doi.org/10.1016/s0014-5793(00)01863-9

Rodriguez-Munoz, A., Aller, E., Jaijo, T., Gonzalez-Garcia, E., Cabrera-Peset, A., Gallego-Pinazo, R., Udaondo, P., Salom, D., Garcia-Garcia, G., & Millan, J. M. (2020). Expanding the Clinical and Molecular Heterogeneity of Nonsyndromic Inherited Retinal Dystrophies. *J Mol Diagn*, *22*(4), 532-543. <https://doi.org/10.1016/j.jmoldx.2020.01.003>

Saito, K., Gotoh, N., Kang, I., Shimada, T., Usui, T., & Terao, C. (2021). A case of retinitis pigmentosa homozygous for a rare CNGA1 causal variant. Sci Rep, 11(1), 4681. https://doi.org/10.1038/s41598-021-84098-9

Sharon, D., Ben-Yosef, T., Goldenberg-Cohen, N., Pras, E., Gradstein, L., Soudry, S., Mezer, E., Zur, D., Abbasi, A. H., Zeitz, C., Cremers, F. P. M., Khan, M. I., Levy, J., Rotenstreich, Y., Birk, O. S., Ehrenberg, M., Leibu, R., Newman, H., Shomron, N.,…Perlman, I. (2020). A nationwide genetic analysis of inherited retinal diseases in Israel as assessed by the Israeli inherited retinal disease consortium (IIRDC). *Hum Mutat*, *41*(1), 140-149. https://doi.org/10.1002/humu.23903

Shuart, N. G., Haitin, Y., Camp, S. S., Black, K. D., & Zagotta, W. N. (2011). Molecular mechanism for 3:1 subunit stoichiometry of rod cyclic nucleotide-gated ion channels. *Nat Commun*, *2*, 457. https://doi.org/10.1038/ncomms1466

Suga, A., Yoshitake, K., Minematsu, N., Tsunoda, K., Fujinami, K., Miyake, Y., Kuniyoshi, K., Hayashi, T., Mizobuchi, K., Ueno, S., Terasaki, H., Kominami, T., Nao, I. N., Mawatari, G., Mizota, A., Shinoda, K., Kondo, M., Kato, K., Sekiryu, T.,…Iwata, T. (2022). Genetic characterization of 1210 Japanese pedigrees with inherited retinal diseases by whole-exome sequencing. *Hum Mutat*, *43*(12), 2251-2264. https://doi.org/10.1002/humu.24492

Sun, Y., Man, J., Wan, Y., Pan, G., Du, L., Li, L., Yang, Y., Qiu, L., Gao, Q., Dan, H., Mao, L., Cheng, Z., Fan, C., Yu, J., Lin, M., Kristiansen, K., Shen, Y., & Wei, X. (2018). Targeted next-generation sequencing as a comprehensive test for Mendelian diseases: a cohort diagnostic study. *Sci Rep*, *8*(1), 11646. https://doi.org/10.1038/s41598-018-30151-z

Villafuerte-de la Cruz, R. A., Garza-Garza, L. A., Garza-Leon, M., Rodriguez-De la Torre, C., Parra-Bernal, C., Vazquez-Camas, I., Ramos-Gonzalez, D., Rangel-Padilla, A., Espino Barros-Palau, A., Nava-Garcia, J., Castillo-Velazquez, J., Castillo-De Leon, E., Del Valle-Penella, A., Valdez-Garcia, J. E., & Rojas-Martinez, A. (2024). Spectrum of variants associated with inherited retinal dystrophies in Northeast Mexico. *BMC Ophthalmol*, *24*(1), 60. https://doi.org/10.1186/s12886-023-03276-7

Villanueva-Mendoza, C., Tuson, M., Apam-Garduno, D., de Castro-Miro, M., Tonda, R., Trotta, J. R., Marfany, G., Valero, R., Cortes-Gonzalez, V., & Gonzalez-Duarte, R. (2021). The Genetic Landscape of Inherited Retinal Diseases in a Mexican Cohort: Genes, Mutations and Phenotypes. *Genes (Basel)*, *12*(11). https://doi.org/10.3390/genes12111824

Weisschuh, N., Obermaier, C. D., Battke, F., Bernd, A., Kuehlewein, L., Nasser, F., Zobor, D., Zrenner, E., Weber, E., Wissinger, B., Biskup, S., Stingl, K., & Kohl, S. (2020). Genetic architecture of inherited retinal degeneration in Germany: A large cohort study from a single diagnostic center over a 9-year period. *Hum Mutat*, *41*(9), 1514-1527. https://doi.org/10.1002/humu.24064

Xue, J., Han, Y., Zeng, W., & Jiang, Y. (2022). Structural mechanisms of assembly, permeation, gating, and pharmacology of native human rod CNG channel. *Neuron*, *110*(1), 86-95 e85. https://doi.org/10.1016/j.neuron.2021.10.006

Zampaglione, E., Kinde, B., Place, E. M., Navarro-Gomez, D., Maher, M., Jamshidi, F., Nassiri, S., Mazzone, J. A., Finn, C., Schlegel, D., Comander, J., Pierce, E. A., & Bujakowska, K. M. (2020). Copy-number variation contributes 9% of pathogenicity in the inherited retinal degenerations. *Genet Med*, *22*(6), 1079-1087. https://doi.org/10.1038/s41436-020-0759-8

Zenteno, J. C., Garcia-Montano, L. A., Cruz-Aguilar, M., Ronquillo, J., Rodas-Serrano, A., Aguilar-Castul, L., Matsui, R., Vencedor-Meraz, C. I., Arce-Gonzalez, R., Graue-Wiechers, F., Gutierrez-Paz, M., Urrea-Victoria, T., de Dios Cuadras, U., & Chacon-Camacho, O. F. (2020). Extensive genic and allelic heterogeneity underlying inherited retinal dystrophies in Mexican patients molecularly analyzed by next-generation sequencing. *Mol Genet Genomic Med*, *8*(1). https://doi.org/10.1002/mgg3.1044

Zhong, H., Lai, J., & Yau, K. W. (2003). Selective heteromeric assembly of cyclic nucleotide-gated channels. *Proc Natl Acad Sci U S A*, *100*(9), 5509-5513. https://doi.org/10.1073/pnas.0931279100

Zhong, H., Molday, L. L., Molday, R. S., & Yau, K. W. (2002). The heteromeric cyclic nucleotide-gated channel adopts a 3A:1B stoichiometry. *Nature*, *420*(6912), 193-198. https://doi.org/10.1038/nature01201
